# Modulation of innate immune response to mRNA vaccination after SARS-CoV-2 infection or sequential vaccination in humans

**DOI:** 10.1172/jci.insight.175401

**Published:** 2024-05-08

**Authors:** Fredrika Hellgren, Anja Rosdahl, Rodrigo Arcoverde Cerveira, Klara Lenart, Sebastian Ols, Yong-Dae Gwon, Seta Kurt, Anna Maria Delis, Gustav Joas, Magnus Evander, Johan Normark, Clas Ahlm, Mattias N.E. Forsell, Sara Cajander, Karin Loré

**Affiliations:** 1 Division of Immunology and Allergy, Department of Medicine Solna, Karolinska Institutet, Stockholm, Sweden & Karolinska University Hospital, Stockholm, Sweden.; 2 Center for Molecular Medicine, Karolinska Institutet, Stockholm, Sweden.; 3 Department of Infectious Diseases and; 4 School of Medical Sciences, Faculty of Medicine and Health, Örebro University, Örebro, Sweden.; 5 Department of Clinical Microbiology, Umeå University, Umeå, Sweden.; 6 Department of Clinical Research Laboratory, Faculty of Medicine and Health, Örebro University, Örebro, Sweden.

**Keywords:** Immunology, Vaccines, Adaptive immunity, Innate immunity

## Abstract

mRNA vaccines are likely to become widely used for the prevention of infectious diseases in the future. Nevertheless, a notable gap exists in mechanistic data, particularly concerning the potential effects of sequential mRNA immunization or preexisting immunity on the early innate immune response triggered by vaccination. In this study, healthy adults, with or without documented prior SARS-CoV-2 infection, were vaccinated with the BNT162b2/Comirnaty mRNA vaccine. Prior infection conferred significantly stronger induction of proinflammatory and type I IFN–related gene signatures, serum cytokines, and monocyte expansion after the prime vaccination. The response to the second vaccination further increased the magnitude of the early innate response in both study groups. The third vaccination did not further increase vaccine-induced inflammation. In vitro stimulation of PBMCs with TLR ligands showed no difference in cytokine responses between groups, or before or after prime vaccination, indicating absence of a trained immunity effect. We observed that levels of preexisting antigen-specific CD4 T cells, antibody, and memory B cells correlated with elements of the early innate response to the first vaccination. Our data thereby indicate that preexisting memory formed by infection may augment the innate immune activation induced by mRNA vaccines.

## Abstract 2

Prior SARS-CoV-2 infection or sequential mRNA vaccination modulates the early innate immune response to mRNA vaccination in humans.

## Introduction

The development and approval of mRNA vaccines against SARS-CoV-2 introduced a new era in vaccinology. mRNA vaccines are capable of inducing robust production of neutralizing antibodies and memory B cells as well as antigen-specific CD4^+^ and CD8^+^ T cells (1–5). However, many of the fundamental mechanisms by which mRNA vaccines induce strong immune responses remain elusive. Notably, mRNA vaccines do not require coadministration with an adjuvant, likely due to their inherent ability to activate strong innate immune responses caused by both the mRNA and the lipid nanoparticle (6). mRNA vaccines stimulate multiple immune pathways, including TLRs and inflammasome activation, leading to production of proinflammatory cytokines and type I IFNs (7–9). Type I IFN signaling is known to suppress protein translation as part of antiviral immune mechanisms (10, 11). It may be inferred that exaggerated TLR signaling induced by mRNA vaccination could cause suppression of protein translation, thereby reducing the amount of vaccine antigen expressed and potentially decreasing immunogenicity. Previous studies have reported both enhancement and detrimental effects by type I IFNs on the antigen-specific immune response induced by mRNA vaccination (12–15). It is, therefore, important to dissect the early activation induced by mRNA vaccines to aid in the identification of optimal stimulation conditions. The interplay of innate and adaptive immune responses to vaccination has been clearly demonstrated in multiple studies, wherein TLR stimulation provided by vaccine adjuvants improved the quality and durability of subsequent adaptive immune responses (16–21). A more complete understanding of the innate immunity triggered by mRNA vaccines would assist in fine-tuning vaccine design to induce sufficient immune activation while simultaneously avoiding unnecessary adverse events.

We and others have previously demonstrated, in nonhuman primate (NHP) models, that mRNA vaccination leads to localized innate immune activation in the injected muscle tissue as well as the lymph nodes that drain the injection site (22–24). This was characterized by infiltration and activation of neutrophils, monocytes, and DCs, as well as detection of type I IFN–related cytokines. Innate immune responses were transient and resolved rapidly, usually within a week after vaccination. However, animal models do not recapitulate all aspects of innate immune activation or side effects reported after mRNA vaccination in humans. Mice and NHPs appear more resistant to side effects by mRNA vaccination, including the exposure to high levels of IFN-α, and appear to produce considerably higher levels of IL-1RA, which dampen IL-1β–driven inflammation (25). Significant innate immune activation has been demonstrated in humans early after mRNA vaccination (26, 27) and may be responsible for several of the clinical side effects reported (28, 29). The portfolio of mRNA vaccines will likely expand to include additional infectious diseases in the coming years, and further investigation of mRNA-induced innate immune reactions in humans is thereby warranted. Notably, there are currently limited data on whether sequential mRNA vaccination influences the degree of innate immune activation, especially for follow-up booster vaccinations after the first 2 doses.

Innate immune responses after mRNA vaccination have been shown to consist of increased frequency of circulating CD14^+^CD16^+^ intermediate (inflammatory) monocytes, IFN-γ secretion in plasma, and transcriptional upregulation of innate and antiviral gene signatures (26, 27). A systems vaccinology approach demonstrated an augmented innate immune response to the second dose of the BNT162b2 SARS-CoV-2 mRNA vaccine, compared with the first dose (27). However, since most individuals were naive to SARS-CoV-2 prior to prime vaccination in this study, the potential influence of preexisting immunity derived from infection was not studied. Additionally, the mechanisms by which the innate response would be augmented by booster vaccination are not well understood. It has been suggested that mRNA vaccines can mediate epigenetic alterations to innate immune cell populations, causing increased responsiveness to stimuli — a phenomenon commonly termed trained immunity (8, 30, 31). Increased innate immune responses to ex vivo stimulation of human PBMC following BNT162b2 mRNA vaccination have been reported (31). This has been proposed to be explained by epigenetic modulatory effects after mRNA vaccination, resulting in increased chromatin accessibility for several type I IFN–related genes (32). However, such changes were short lived and only detectable for 28 days. In contrast, others found no evidence of trained immunity in BNT162b2-vaccinated study participants when testing PBMCs sampled at 28 days (33). The potential manifestation or absence of trained immunity after mRNA vaccination may be influenced by timing of sampling, type of vaccine, previous exposures, or characteristics of vaccine recipients and, therefore, warrants further study.

An alternate hypothesis of the enhanced innate response after repeated mRNA vaccination is that the presence of SARS-CoV-2–specific antibodies at the boost immunization may modulate innate immune cell activation, as has been observed for other vaccine modalities (34). A role for IFN-γ in regulating the innate immune activation induced by mRNA vaccination was described in mice, demonstrating that blocking IFN-γ signaling reduced CD86 expression on several cell subsets as well as reduced induction of proinflammatory genes in monocytes (3). This suggests that preexisting T cell immunity can play a role in regulating innate immune activation by mRNA vaccination. Whether there is any effect on vaccine-induced innate responses by preexisting immunity to SARS-CoV-2 that results from prior infection, not vaccination, has not been fully described.

Here, we established a clinical study with a primary focus on conducting thorough analyses of the innate immune responses following each of 3 doses of mRNA vaccination within the same cohort of individuals. The initiation of this study coincided with the early rollout of SARS-CoV-2 vaccines, during a period when social restrictions were still in place. This timing allowed us to enroll both an immunologically SARS-CoV-2–naive group and a group that had experienced the infection once. The limited well-characterized cohort facilitated comprehensive assessment of various aspects of innate immunity, including transcriptomics, cell differentiation, and the secretion of chemokines and cytokines. By longitudinally monitoring these individuals, we were able to observe how their responses evolved over time and enabled the evaluation of trained immunity both before and after vaccination within the same individuals.

## Results

### Three doses of mRNA vaccine are needed in infection-naive individuals to confer similar quantity and quality of antibodies as in infection-experienced individuals.

**Figure 1:**
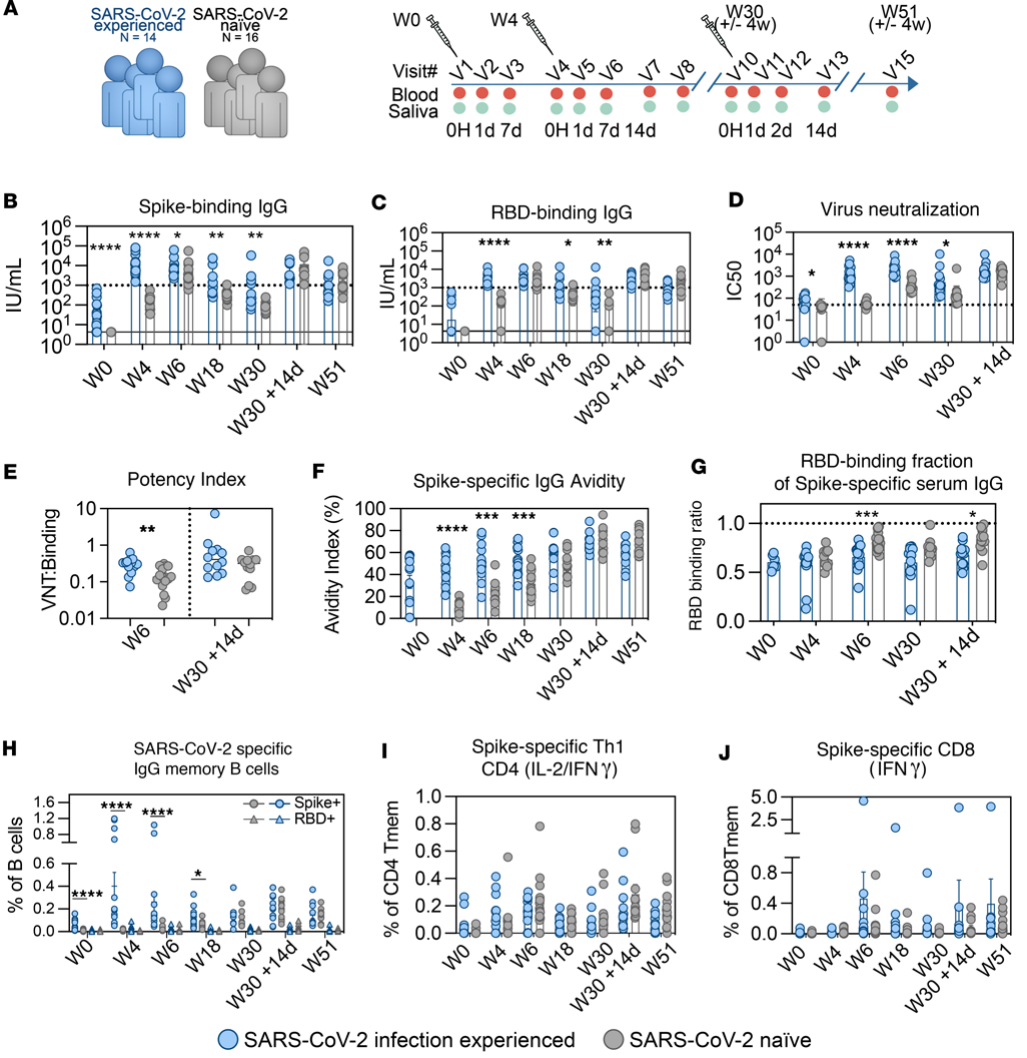
Three doses of vaccine required to attain similar levels of humoral immunity in SARS-CoV-2 infection naive as infection-experienced individuals. (**A**) Schematic of group division and sampling schedule. (**B** and **C**) Spike-binding (**B**) and RBD-binding (**C**) IgG plasma titers, quantified by binding ELISA. Data reported as IU/mL based on the WHO First International Standard. Data are shown as geometric mean ± geometric SD. (**D**) Plasma live virus neutralizing titers (Wu-Hu-1 equivalent strain, Swedish isolate). Data are shown as geometric mean ± geometric SD. (**E**) Antibody potency index, calculated as ratio of virus neutralizing titer (IC_50_) to spike binding titer (IU/mL). Line indicates geometric mean. (**F**) Spike IgG antibody avidity measured by chaotropic wash ELISA, reported as avidity index (% of antibody binding remaining after chaotropic wash). Data are shown as mean ± SEM. (**G**) Fraction of RBD-binding plasma IgG out of total spike binding measured by competition ELISA using recombinant RBD in solution. Dotted line indicates binding ratio of 1.0. Data are shown as mean ± SEM. (**H**) Fractions of total spike-specific and spike/RBD-specific IgG^+^ B cells over time, shown as percentage of total B cells. Representative gating of total spike and spike/RBD-specific B cells shown in Supplemental Figure 14. Data are shown as mean ± SEM. (**I**) Spike-specific CD4 T cells producing IFN-γ and/or IL-2 in response to SARS-CoV-2 spike overlapping peptide stimulation. Data shown as percentage of CD4 memory T cells. Data are shown as mean ± SEM. (**J**) Spike-specific CD8 T cells producing IFN-γ in response to SARS-CoV-2 spike overlapping peptide stimulation. Data are shown as percentage of CD8 memory T cells. Data are shown as mean ± SEM. Groups were compared by multiple Mann-Whitney *U* test with comparison between groups at each time point and *P* value adjustment using the Holm-Šidák method (α threshold 0.05). Number of participants analyzed: Week 0 = 30; Week 4 = 30 (**A**–**G**), 28 (**H**–**J**); Week 6 = 29; Week 18 = 29; Week 30 = 23; Week 30 + 14d = 24; Week 51 = 28 (**B**–**G**, **I**, and **J**), 27 (**H**). The *x* axis indicates time point.

We utilized the SARS-CoV-2 vaccination program of multiple doses of mRNA vaccine administered to study the characteristics of the early innate immune activation induced shortly after vaccination. The study cohort characteristics at study start are summarized in Table 1. Healthy adults, either naive or with prior SARS-CoV-2 infection, were vaccinated with 2 doses of the BNT162b2/Comirnaty mRNA vaccine 4 weeks apart, followed by a third dose given 6–8 months thereafter (Figure 1A). The history of SARS-CoV-2 infection prior to the first vaccination was PCR confirmed in all but 1 participant, for whom infection was instead confirmed by an antibody test. All individuals in the SARS-CoV-2–experienced group reported mild to moderate symptoms, except 1 participant who reported severe symptoms requiring hospitalization (Table 2).

Prime immunization induced IgG binding titers against both the SARS-CoV-2 full spike protein and the spike receptor binding domain (RBD) in all SARS-CoV-2–experienced individuals as well as in a majority of naive individuals. In line with prior data (35), the infection-experienced group displayed significantly higher levels of binding antibody to spike (Figure 1B) and RBD (Figure 1C) after the first dose. Following the second dose, the naive group showed a robust boosting effect, while titers in the experienced group did not further increase significantly (Supplemental Figure 1, A and B; supplemental material available online with this article; https://doi.org/10.1172/jci.insight.175401DS1). Following the second dose, SARS-CoV-2–experienced individuals retained higher neutralizing titers (SARS-CoV-2/01/human/2020/SWE) (Figure 1D) and neutralization potency — i.e., the ratio between binding and neutralization (Figure 1E) — indicating that there were persisting differences between groups in the quality of antibodies despite similar titers.

In line with these data, spike-specific IgG binding avidity was markedly higher in infection-experienced study participants after both the first and second vaccination compared with naive individuals (Figure 1F). The proportion of the antibody response targeted to the spike RBD was slightly higher in the naive group (Figure 1G). Antibody titers waned during the 5- to 7-month period between the second and third dose in both groups. The third immunization effectively boosted the titers in both naive and experienced study participants (Figure 1, B and C) and equalized both neutralization potency (Figure 1E) and IgG avidity to spike (Figure 1F) between study groups. Three doses of the mRNA vaccine were, therefore, needed to reach similar quantity and quality of antibodies in naive individuals as compared with individuals having preexisting immunity from prior infection. Additionally, higher levels of IgA were observed in the plasma of SARS-CoV-2 infection–experienced participants after the first dose (Supplemental Figure 1C). Assessment of antibodies in saliva after 2 vaccine doses showed similar titers of IgG (Supplemental Figure 1D) but persisting differences in IgA (Supplemental Figure 1E).

In line with the antibody responses, 3 doses were required to reach the same frequencies of spike- or RBD-specific memory B cells between the groups (Figure 1H). The low or undetectable numbers of spike-specific B cells in the experienced group at study start were boosted efficiently by the prime mRNA vaccination. The infection-naive group developed detectable spike-specific memory B cells in response to primary vaccination but required a third dose to reach similar levels as the infection-experienced cohort. Spike-specific CD4^+^ T cells were low or undetectable by antigen recall assay in both the infection-experienced and naive study groups at study start. The CD4^+^ memory response to vaccination was dominated by IFN-γ and IL-2, indicating a Th1-skewed immune profile, as has been previously demonstrated for mRNA vaccines (1) (Figure 1I and Supplemental Figure 2, A–F). Spike-specific CD4^+^ memory T cell responses followed similar kinetics and reached similar levels in both study groups after mRNA vaccination (Figure 1I). Spike-specific CD8^+^ memory T cells also developed in response to mRNA vaccination but were generally less detectable compared with CD4^+^ T cells, with no significant difference between the study groups (Figure 1J and Supplemental Figure 3, A–E). In summary, a third dose was required in order to boost antibody titers, as well as B cell memory, to comparable levels in an infection-naive group compared with infection-experienced individuals.

### SARS-CoV-2 infection–experienced individuals show increased proinflammatory transcriptional changes in response to first mRNA vaccination, compared with infection-naive group.

Although adaptive immunity to BNT162b2 has been characterized extensively, questions remain regarding the innate immune response to mRNA in humans. Whether innate immune responses change upon repeated mRNA vaccination, or preexisting immunity from infection, has not been fully elucidated. In addition, the link between the early innate responses and the quality of subsequent adaptive immunity remains incompletely understood. This study was, therefore, designed to focus on in-depth mapping of early innate responses.

**Figure 2:**
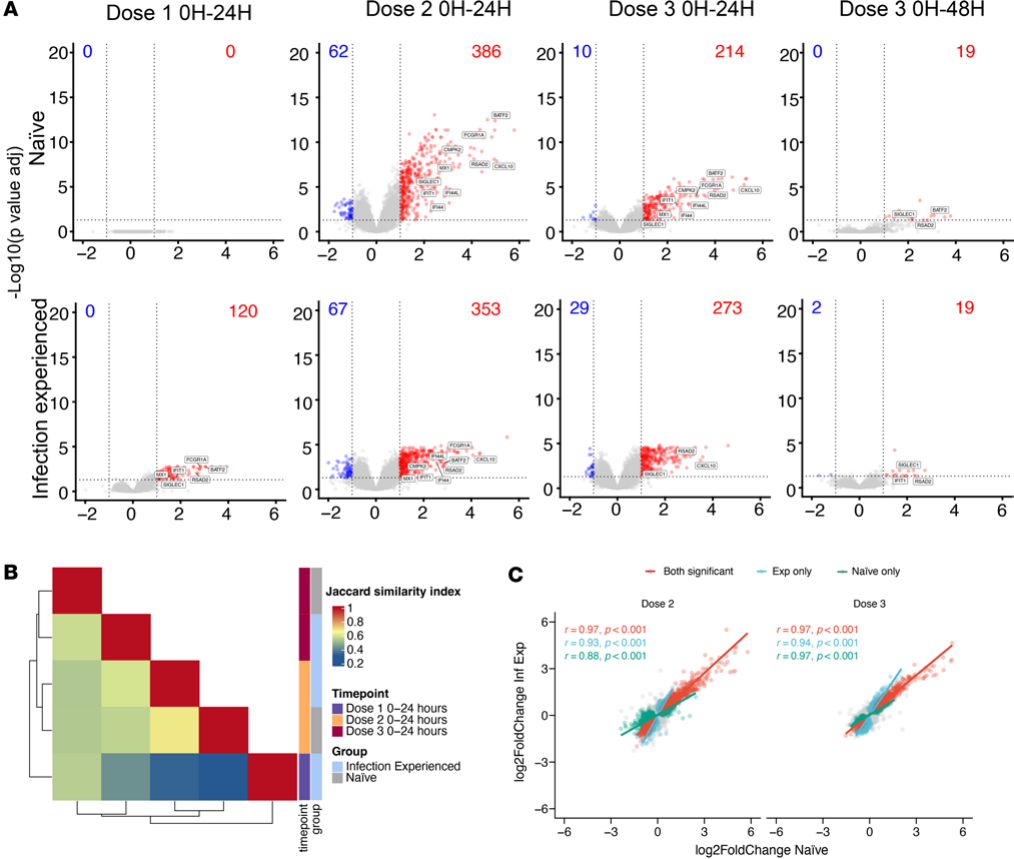
Increased level of differential gene expression in infection-experienced participants compared with infection-naive in response to first mRNA vaccination. (**A**) Volcano plots displaying differentially regulated genes in peripheral blood measured by RNA-Seq. Fold changes and *P* values for each sample group generated by Wald test between prevaccination (0 hours [0H]) and postvaccination samples (24H or 48H) at each vaccine dose. Total number of differentially up- or downregulated genes are indicated in each plot. Cut-off for significant differential regulation were log_2_(fold change) > 1, FDR-adjusted *P* < 0.05. (**B**) Heatmap displaying overlap (Jaccard index/Jaccard similarity coefficient) between differentially regulated genes compared between study groups and time point. (**C**) Pearson’s correlation of fold changes in individual genes identified as differentially regulated in any group. *n* = 15 (dose 1); 14 (dose 2); 13 (dose 3) (Supplemental Figure 4B).

Whole blood RNA-Seq was performed for a subset of study participants (*n* = 15). Samples were taken directly prior to each vaccination, and 24 or 48 hours afterward. Transcriptomic profiling revealed a greater number of differentially expressed genes (DEGs) following the prime vaccination in infection-experienced compared with naive individuals (Figure 2A and Supplemental Figure 4). In contrast, at the first boost immunization, where naive individuals had also developed immunity to the vaccine antigen, the number of DEGs was substantially enhanced in the naive group and was overall similar between the 2 study groups. The third dose, given 6–8 months following study start, induced similar numbers of DEGs between study groups (Figure 2A and Supplemental Figure 4). Examination of the blood transcriptome at 48 hours after the third vaccination (relative to prevaccination samples) demonstrated a decrease in the number of DEGs compared with what was seen at 24 hours, indicating that most vaccine-induced transcriptional changes are short lived (Figure 2A and Supplemental Figure 4). Regarding specific genes regulated, there was a high degree of overlap between the study groups, indicating that the innate immune response to vaccination was phenotypically similar across the groups (Figure 2B). There was a notable correlation in DEG fold changes between groups, indicating that, while certain genes reached statistical significance for differential expression in one group, there was still a discernible degree of change occurring in the other group (Figure 2C). Among genes categorized as differentially expressed after vaccination (with an absolute fold change > 2 and adjusted *P* < 0.05), upregulation was observed for type I IFN–inducible genes RSAD2, IFIT1, and CXCL10, along with the monocyte-macrophage differentiation–associated gene CMPK2 (Figure 2A).

Prevaccination samples from experienced and naive individuals showed overall similar baseline gene expression profiles. No differences were observed that clearly explained the difference in innate immune response to the prime vaccination (Supplemental Figure 5, A–C). Comparison of samples taken on the day of each vaccination did not reveal significant differences over time, demonstrating that no significant persisting transcriptional changes were induced by mRNA vaccination (Supplemental Figure 5, D–G).

**Figure 3:**
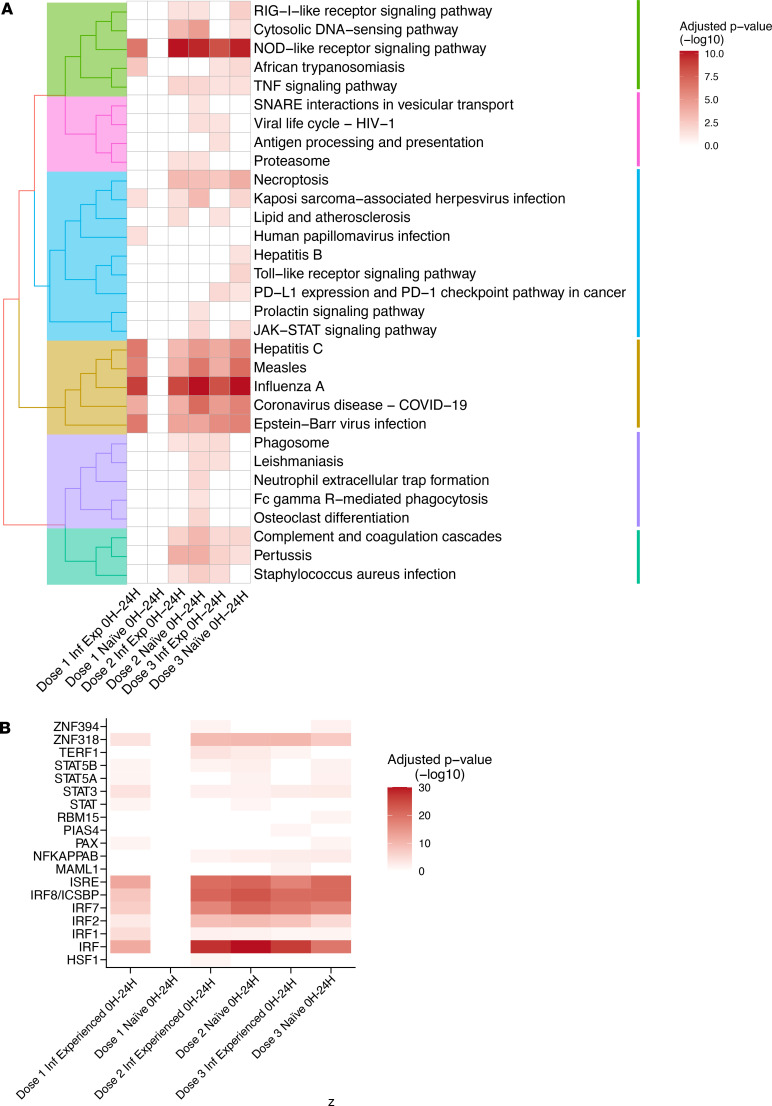
Genes differentially upregulated in response to mRNA vaccination are enriched for antiviral and proinflammatory signaling pathways. (**A**) Overrepresentation analysis of DEGs with log_2_(fold change) greater than 1 using KEGG Pathway database. (**B**) Overrepresentation analysis of DEGs with log_2_(fold change) greater than 1 using the MSigDB subset of Transcriptional Factor Targets (C3: TFT). *P* value cut-off for consideration of genes as differentially expressed/DEGs was FDR-adjusted *P* < 0.05. *n* = 15 (dose 1); 14 (dose 2); 13 (dose 3).

**Figure 4:**
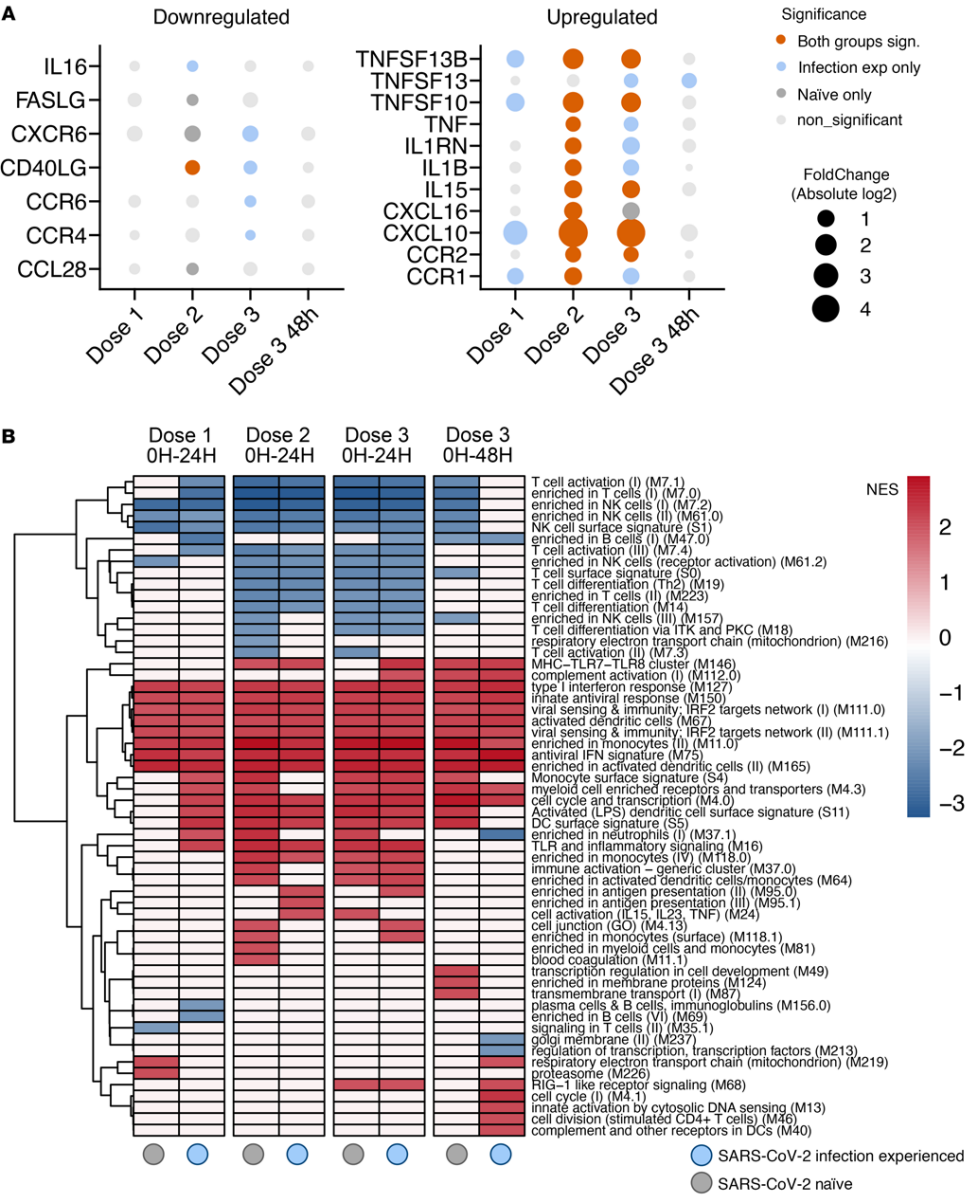
Transient type I IFN polarized transcriptional changes following mRNA vaccination. (**A**) Collection of significant DEGs of immune-associated groups of receptor ligands and receptors annotated in the HUGO database (total of 157). The bubble size represents the mean of absolute fold change when significant for both groups but represents the value itself when significant for only 1 of the groups. *P* value cut-off for consideration of genes, as differentially expressed/DEGs was FDR-adjusted *P* < 0.05. (**B**) Gene set enrichment analysis based on fold change ranking using previously described blood transcription modules (36). Gene modules with absolute normalized enrichment score (NES) > 2 are shown. *n* = 15 (dose 1); 14 (dose 2); 13 (dose 3).

Overrepresentation analysis of genes significantly upregulated in response to mRNA vaccination, using the Kyoto Encyclopedia of Genes and Genomes (KEGG) pathway database, identified functional clusters associated with several viral infections, indicating activation of antiviral cellular programming (Figure 3A). At the second and third doses, enrichment was also seen for the NOD-like receptor, RIG-I–like receptor, and TNF signaling pathways (Figure 3A). Overrepresentation analysis of DEGs using the MSigDB subset of Transcriptional Factor Targets (C3: TFT) found enrichment for targets of several members of the IFN regulatory transcription factor (IRF) and signal transducer and activator of transcription (STAT) families (Figure 3B), indicating induction of IFN signaling. Targeted analysis of cyto-/chemokines and their receptors identified significant upregulation of CXCL10, IL-15, and IL-1B and several TNF superfamily member genes in response to mRNA vaccination, particularly doses 2 and 3. CD40LG was seen significantly downregulated in both groups at second vaccination (Figure 4A).

Gene set enrichment analysis (GSEA) using previously defined blood transcription modules (36) demonstrated induction of gene sets associated to type I IFN signaling, antigen presentation, and innate immune activation, similar to previous results (27). The functional profile of gene set enrichment was similar between the 2 groups and across all 3 doses (Figure 4B), demonstrating that the lower activation to the prime immunization in naive individuals was of a similar profile but less robust and that the phenotypic characteristics of the innate immune response to the mRNA vaccine were not substantially altered by either SARS-CoV-2 infection or sequential immunizations. In summary, we observed a transient type I IFN–skewed inflammatory response to mRNA vaccination that was augmented in study participants with prior infection in response to the first vaccine dose.

### Infection-experienced study participants show elevated proinflammatory cytokine and chemotactic responses to mRNA vaccination, as compared with the infection naive group after prime, but not boost, vaccinations.

**Figure 5:**
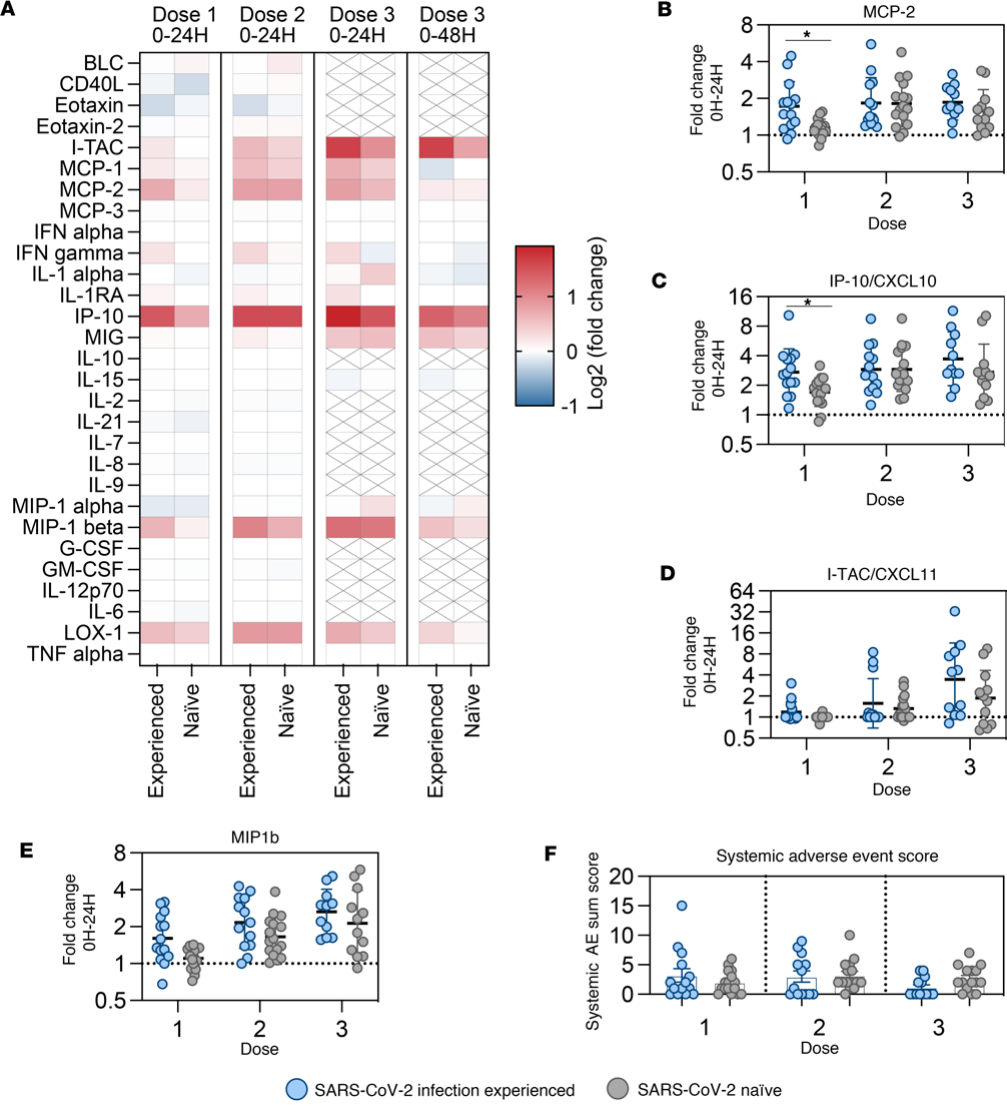
Transient increases in serum levels of proinflammatory cytokines following mRNA vaccination. (**A**) Summary of changes in serum cytokines measured by Luminex across vaccine doses, reported log_2_-fold change calculated between day of vaccination and 24H or 48H after vaccination. Crossed-through boxes indicate cytokines not tested at dose 3. (**B**–**E**) Detection of selected cytokines in serum before and after mRNA vaccination by Luminex bead–based multiplex assay. Shown as fold change at 24H compared with 0H at each vaccine dose. Data are shown as geometric mean ± geometric SD. The *x* axis labels denote vaccine dose numbers. (**F**) Scoring of systemic adverse events per study participant and vaccine dose. Systemic adverse events (AE) were classified by the following system: 0 = local AE < 48H; 1 = local AE > 48H; 2 = systemic < 48H; 3 = systemic > 48H. Systemic AE scores were summed for each individual and vaccine dose. Data are shown as mean ± SEM. Groups were compared by multiple Mann-Whitney *U* test with comparison between groups at each time point and *P* value adjustment using the Holm-Šidák method (α threshold 0.05). Number of study participants shown (all panels): dose 1 = 30; dose 2 = 29; dose 3 = 23.

In line with whole blood transcriptomic data, we observed transient secretion of several proinflammatory cytokines and chemokines 24 hours following mRNA vaccination (Figure 5A). Notably increased cytokines in serum included monocyte chemoattractant protein 2 (MCP-2) (Figure 5B), IFN-inducible IP-10/CXCL-10 (Figure 5C), I-TAC/CXCL-11 (Figure 5D), and MIP-1b (Figure 5E). Increases in serum cytokine concentrations were overall transient and returned to baseline levels within 7–14 days (Supplemental Figure 7, A–D). Although there was a clear induction of IFN-inducible cytokines, IFN-α did not reach detectable levels in serum (Supplemental Figure 7E).

Similar to transcriptomic analyses, induction of MCP-2 (Figure 5B) and CXCL10 (Figure 5C) was more pronounced in infection-experienced study participants compared with the naive group after the prime vaccination. The second and third vaccinations induced similar cytokine secretion in both study groups (Figure 5, A–E). Notably, although we observed both a stronger upregulation of genes and increased production of several proinflammatory cytokines in the experienced group at the prime immunization, there was no clear increase in occurrence of systemic adverse events in this group (Figure 5F).

### mRNA vaccination induces a rapid and transient increase in proportion of monocytes in circulation.

**Figure 6:**
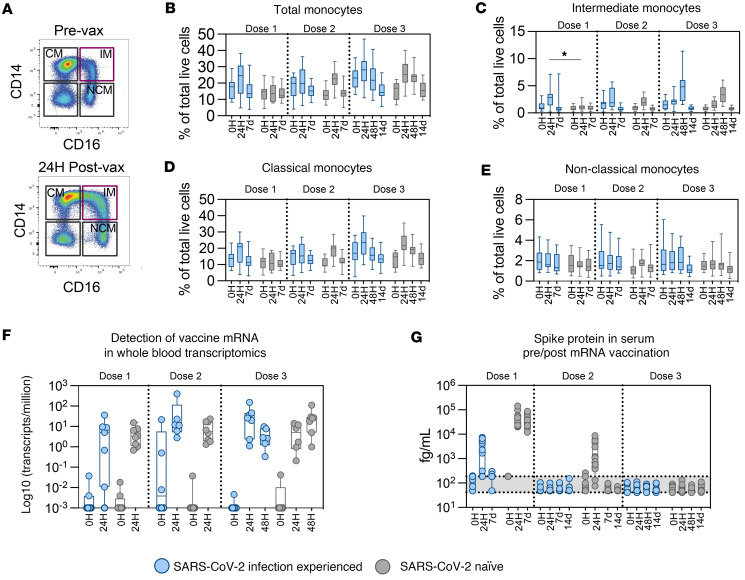
Transient changes in distribution of immune cell populations in peripheral blood after mRNA vaccination. (**A**) Representative gating of monocyte subsets in peripheral blood. (**B**–**E**) Quantification of monocyte and monocyte subsets (CM, classical; IM, intermediate; NCM, nonclassical) as proportions of total gated live single cells per sample. (**F**) Detection of mRNA vaccine transcripts in bulk blood transcriptomic data. The vaccine mRNA sequence was sourced as reported in the repository by Jeong et al. (39) and appended to the human reference genome prior to alignment of blood RNA-Seq data. Transcripts matching the reported BNT162b2 vaccine sequence are reported as log(transcripts per million). (**G**) Detection of spike protein in serum before and after mRNA vaccination by Mesoscale Discovery assay. Box and whiskers indicate minimum to maximum. Gray shading indicates lower limits of detection (range of total 4 MSD plates run). Groups were compared by multiple Mann-Whitney *U* test with comparison between groups at each time point and *P* value adjustment using the Holm-Šidák method (α threshold 0.05). No statistical tests performed for **F** and **G**. Number of participants shown: dose 1 = 29, dose 2 = 28 (0H = 27), and dose 3 = 23 (0H = 19) (**B**–**E**); dose 1 = 15, dose 2 = 14, and dose 3 = 13 (**F**); and dose 1 = 30; dose 2 = 29; dose 3 = 23 (**G**).

Apart from gene modulation and cytokine secretion, we observed an increase in the proportion of circulating total monocytes in peripheral blood shortly after mRNA vaccination (Figure 6B and Supplemental Figure 10A), as previously described (27). The proportional increase in monocytes was mainly composed of classical CD14^+^ (Figure 6C and Supplemental Figure 10C) and intermediate CD14^+^CD16^+^ monocytes (Figure 6D and Supplemental Figure 10B). Expansion of intermediate monocytes in response to vaccination with mRNA or TLR-stimulating vaccine adjuvants has been previously described by us and others in both NHP models and humans (17, 20, 23, 27, 37, 38). Similarly to gene modulation and cytokine secretion, a trend toward higher levels of both total, intermediate, and classical (Figure 6) monocytes were evident in infection-experienced vaccinees compared with the naive group after the first, but not second or third, immunizations. Transient decreases in the fraction of CD11c^+^ conventional DCs (cDCs) were also observed at 24 hours after mRNA vaccination compared with steady state (Supplemental Figure 9B). In terms of lymphoid cell populations, we observed decreased proportions of circulating lymphocytes at 24 hours after vaccination (Supplemental Figure 9, D–F), possibly indicating exit of T cells, B cells, and CD16^+^ NK cells from circulation, with potential migration to secondary lymphoid tissues.

### Transient spike protein and mRNA vaccine sequence found systemically following immunization.

To estimate the duration of the presence of mRNA vaccine or in vivo production of spike protein following vaccination, we searched for the vaccine RNA-Seq in the transcriptomics data set from blood and measured the amount of spike protein present in serum at different time points after all 3 immunizations. We detected the mRNA vaccine sequence (sequence reported as corresponding to the BNT162b2 vaccine in the repository by Jeong et al.; ref. 39) at 24 hours after each vaccination, with lower or no detection evident at the days of vaccination (Figure 6F). This indicated that the mRNA vaccine can appear in the circulation early after immunization. RNA-Seq for the native virus spike protein and the virus nucleocapsid protein were not readily detectable in blood RNA-Seq data at any time point, solidifying the specificity of the results (Supplemental Figure 11, A–C). In addition, spike protein was readily detectable at 24 hours and remained in the serum up to 7 days after vaccination but was cleared within 4 weeks (Figure 6G). We observed lower levels of spike protein detectable in serum of the individuals from the experienced group, and this was likely due to the presence of spike-specific antibodies interfering with spike detection, as demonstrated earlier (40). As expected under the assumption that antibody masking was present, we observed that detected levels of spike protein declined with each immunization to virtually undetectable in both groups after the third vaccination (Figure 6G). Collectively, this demonstrates that both the mRNA vaccine and spike produced by vaccination enter the circulation early after immunization but disappear within 4 weeks.

### In vitro TLR stimulation does not indicate trained immunity induced by prior SARS-CoV-2 infection or mRNA vaccination.

**Figure 7:**
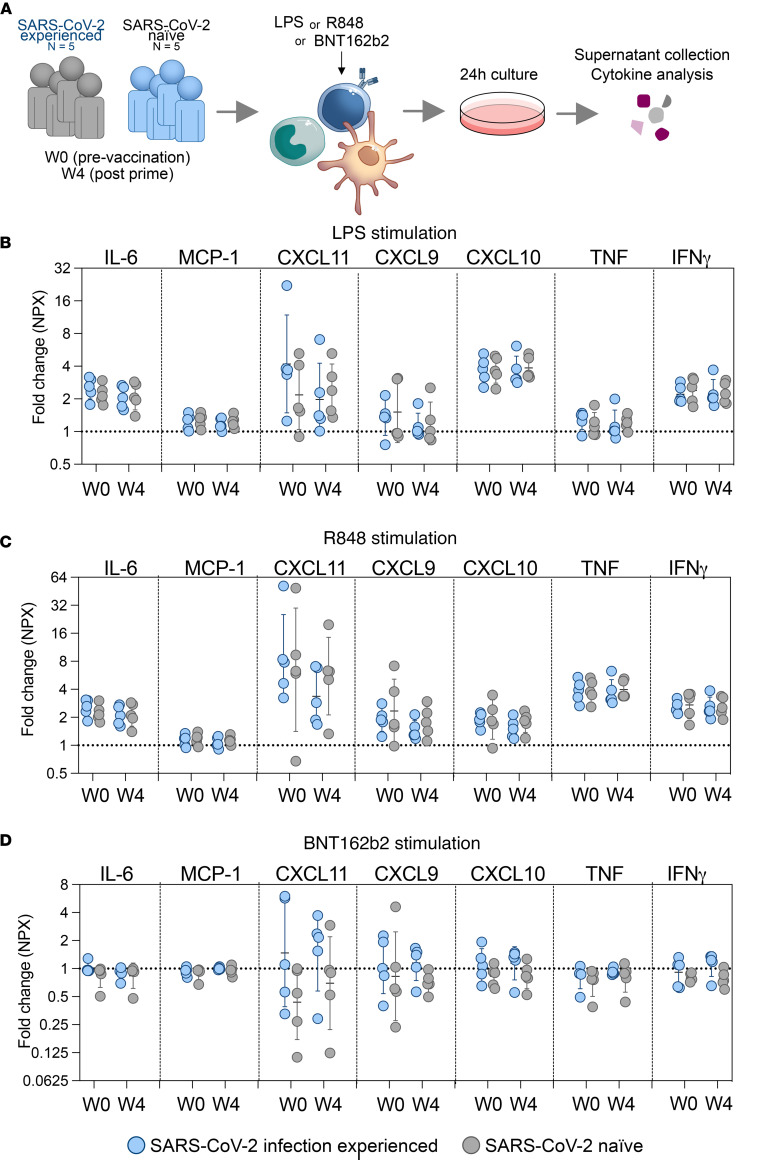
Assessment of general reactivity to TLR ligands before and after mRNA vaccination. (**A**) Schematic of assay principle. (**B**–**D**) Fold changes of selected cytokines (out of total 92 analytes) after stimulation with LPS (**B**), R848 (**C**), or BNT162b2 (**D**). Cytokine secretion was measured in culture supernatants by OLINK proximity extension assay and fold changes calculated based on unstimulated controls (cultured with R10 media only). Groups were compared by multiple Mann-Whitney *U* test with comparison between groups at each time point and *P* value adjustment using the Holm-Šidák method (α threshold 0.05). Dotted line denotes fold change 1.0 (i.e., no change). *n*= 10 (5 per study group).

The concept of trained immunity where innate immune cells acquire memory and exhibit enhanced responses to previously encountered infectious agents or subsequent stimuli has been proposed to occur after mRNA vaccination (8, 30, 41). We did not detect substantial increases in serum levels of IL-6 or TNF, the classical cytokines associated with trained immunity, after mRNA vaccination in either study group, irrespective of vaccine dose (Figure 5A). To address whether cells from individuals in the experienced group demonstrated enhanced responses to stimuli, we performed in vitro cultures of PBMCs collected directly prior to the first 2 vaccinations in the presence of the TLR7/8 ligand R848, TLR4 ligand LPS, or the mRNA vaccine itself (Figure 7A). Cell culture supernatants were analyzed for several proinflammatory cytokines. While stimulation with TLR ligands induced significant cytokine production, no difference in secretion of TNF or IL-6 was observed between cells from the experienced compared with naive group (Figure 7, B–D). Secretion of MCP-1; CXCL9, -10, or -11; or IFN-γ was also similar between study groups. We did not observe that prior SARS-CoV-2 infection or sequential mRNA vaccination altered the overall responsiveness to TLR stimulation. These results suggest that the enhanced innate vaccine responses observed after infection or sequential vaccination were not due to a generalized training effect on the innate immune system.

### Elements of preexisting adaptive immunity correlate with increased innate immune response to prime vaccination in infection-experienced individuals.

**Figure 8:**
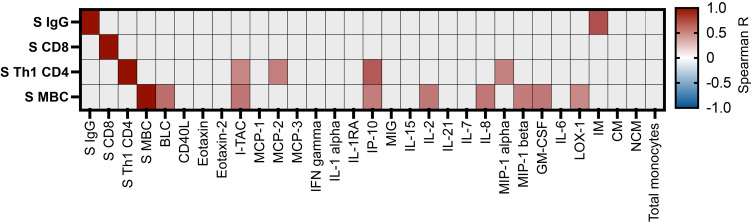
Correlation of selected innate immune parameters and humoral immune responses to vaccination at first vaccine dose. Correlation matrix displaying relationship between selected preexisting adaptive immune parameters measured at study start, and fold changes in innate immune parameters from study start to 24 hours after first mRNA vaccination. Nonparametric Spearman correlation. Color scale denotes Spearman *R* value. Correlations with *P* < 0.05 were considered significant. Nonsignificant correlations shown in gray. *n* = 14. Only SARS-CoV-2 infection–experienced study group included in analysis.

We aimed to identify aspects of the preexisting adaptive immune response to SARS-CoV-2 that could be associated with a more pronounced early innate immune response to prime vaccination. We found that, within the infection-experienced cohort, preexisting levels of spike-specific CD4 memory T cells and spike-specific memory B cells were significantly correlated to the fold change of several proinflammatory cytokines, notably the IFN-inducible cytokines CXCL10 and CXCL11, at 24 hours after the first vaccine dose (Figure 8). The level of spike-specific IgG in plasma was significantly correlated to the fold change in differentiation of intermediate monocytes at 24 hours (Figure 8). Taken together, these data indicate that preexisting adaptive immunity may contribute to enhancement of the innate immune response to mRNA vaccination.

## Discussion

Despite the proven efficacy of mRNA vaccines, questions remain regarding the characteristics and regulation of the innate immune response to mRNA vaccination. mRNA vaccines possess an intrinsic capability to elicit robust innate immune responses, attributed to both the mRNA and the lipid nanoparticle. We and others have previously characterized the innate immune response to mRNA vaccines in NHP models, with key features including systemic monocyte differentiation toward an intermediate or inflammatory phenotype and the secretion of type I IFN–related cytokines as well as MCP in the serum (22–24). In this study, we were able to confirm that the innate immune response to mRNA vaccination in humans closely mirrors responses in NHPs. Although the magnitude of vaccine-induced inflammation is generally lower in humans compared with NHPs, phenotypic features are highly similar. This signature of innate immunity also aligns with seminal work characterizing human responses to SARS-CoV-2 mRNA vaccination early during the pandemic (27). This study included almost exclusively SARS-CoV-2–naive individuals and demonstrated augmented IFN-γ production after the second vaccine dose compared with the first, in line with our findings in the naive group. We additionally observed transient decreases in proportions of circulating lymphocytes at 24 hours after vaccination compared with baseline; this may indicate trafficking of these cells to the site of injection and draining lymph nodes.

Our assessments of the early transcriptomic changes, plasma cytokine levels, and fluctuations in circulating innate cell populations collectively indicate a stronger inflammatory response to mRNA vaccination in participants with a documented prior SARS-CoV-2 infection compared with infection-naive individuals. Several possible explanations for this can be hypothesized. It has been suggested that SARS-CoV-2 infection can cause long-term modulation of innate immune reactivity, termed “trained immunity” as mentioned above. Single-cell ATAC-Seq demonstrated that recovered patients with COVID-19 showed differential epigenetic programming of several immune cell populations as compared with healthy donors. Monocytes appeared to be in a heightened state of activation, and B cells more readily differentiated from the naive state to antibody-secreting cells (42). We did not observe significant differences between infection-experienced and naive study participants in circulating innate cell populations or expression of activation markers at study start. In line with phenotypic data, we also did not detect significant group differences in baseline gene expression, although we acknowledge that whole blood RNA-Seq may provide limited ability to detect subtle changes confined to select cell subsets. Since we did not examine changes in chromatin accessibility in our study participants, we cannot exclude the possibility that epigenetic reprogramming occurred in our SARS-CoV-2–experienced cohort. However, the lack of functional differences in the PBMCs from the groups to produce cytokines in response to multiple stimuli reinforces that an immune training effect did not primarily contribute to the increased early inflammatory response to mRNA vaccination in the infection-experienced group.

Another possible explanation for the augmented innate immune response in SARS-CoV-2 infection–experienced study participants is the influence of preexisting immunity, such as formation of immune complexes with SARS-CoV-2–specific antibodies. It was previously shown that antigen-antibody complexes can augment the migration of DCs from peripheral tissues to lymph nodes in a mouse model (43). Using the NHP model, we also observed that preexisting immunity influenced the infiltration and activation of cells at the site of vaccination (34). In the current study, we found that frequencies of preexisting spike-specific CD4^+^ memory T cells, as well as spike-specific memory B cells, significantly correlated with the levels of several proinflammatory cytokines induced in response to the first mRNA vaccination in the infection-experienced group. Levels of preexisting spike-specific IgG correlated with the fold change in circulating CD14^+^CD16^+^ intermediate monocytes. These findings would indicate that preexisting adaptive immunity can influence the early inflammatory response to mRNA vaccination. The increased innate response to the second vaccination compared with the first in naive individuals may, therefore, be antigen dependent and involve antigen-specific immune cells or antibodies. However, further studies are needed to elucidate the mechanisms by which adaptive immunity may modulate the early innate responses to vaccines and to elucidate to what extent this effect is dependent upon the antigen that the mRNA encodes.

It also remains to be investigated whether the increase in the innate immune responses from prime to boost vaccination in naive individuals is mediated by the same mechanism that augments innate immune activation to the first dose in infection-experienced individuals. The level of preexisting adaptive immunity was demonstrated to affect innate immune reactivity to Omicron infection in SARS-CoV-2–vaccinated and unvaccinated individuals (44). The comparability of these data to our study may be limited, as inhibitory effect of preexisting immunity on viral replication may mean that adaptive-innate interactions are not identical in infection and vaccination contexts. However, the data obtained in ref. 44 underscore the regulatory role of immunological memory in influencing innate immune reactivity. Importantly, our study shows that a third immunization did not appear to further augment the innate inflammation induced by the mRNA vaccine compared with the second dose. This observation is important, given the potential for mRNA vaccines to become more widely used in the future.

Our study has limitations of a relatively small sample size, which restricted our ability to definitively assess the effect of factors such as age and comorbidities on the innate immune response to vaccination. Additionally, very few individuals in our SARS-CoV-2–infected group experienced moderate or severe COVID-19 disease. Hence, we could not draw conclusions on the effect of disease severity on the modulation of mRNA vaccine–induced innate activation. These questions require further studies, particularly given that the use of mRNA vaccines is likely to increase in the coming years.

## Methods

### Sex as a biological variable.

Both male and female participants were included in the study population. There was a bias toward higher inclusion of female participants (Table 1). The ratio of female to male participants was similar between the 2 study groups (prior SARS-CoV-2 infection and SARS-CoV-2); thus, we do not expect an influence on the outcomes of the study in terms of group comparisons. The findings of the study are expected to be relevant for both sexes.

### Study population.

Study participants were recruited among health care workers at the Örebro Universitetssjukhus/Örebro University Hospital. The first vaccine dose was given in April–May 2021. Study participants were sampled adjacent to each vaccine dose according to the schedule shown in Figure 1A. One study participant did not receive dose 2 within the defined time frame (data not shown beyond day 7 of prime vaccination [noted as V3 in Figure 1A]). One study participant received dose 3 substantially later than the defined time frame (adaptive immune data not shown for V15). One study participant was lost to follow-up (no data collected beyond V8). Self-reported adverse events were registered at each follow-up visit. Numbers of study participants shown for each assay are reported in figure legends.

### SARS-CoV-2 infection history.

The SARS-CoV-2 infection–experienced group was recruited based on self-reported infections that occurred between March 2020 and January 2021. All recorded infections were PCR verified except for one that was confirmed by follow-up serology only. Only 1 study participant required hospitalization due to COVID-19. One study participant was originally enrolled as infection experienced, despite having an inconclusive diagnostic PCR result. Preliminary analysis of the antibody response to the first vaccination showed antibody kinetics that were in line with those of the SARS-CoV-2–naive group. Repeat of the diagnostic PCR test using frozen stored swab material yielded a negative test result. The study participant was, therefore, reclassified as naive at study start. The possibility of incidences of asymptomatic abortive infection not resulting in seroconversion, as has been reported (45, 46), cannot be ruled out in the naive group. Data on SARS-CoV-2 breakthrough infections were self-reported at the 12-month follow-up and are shown in Supplemental Figure 12. At all other instances, study participants are classified according to SARS-CoV-2 infection history at study start. All reported breakthrough infections except one occurred after the third vaccination. No significant differences in the magnitude of spike-specific adaptive responses were observed at the 12-month time point, when study participants were grouped by breakthrough infection status (Supplemental Figure 12, B–E), though a trend toward slightly elevated Th1-type CD4 responses was seen (Supplemental Figure 12D).

### Sample processing.

Blood was collected into Cell Prep Tubes Vacutainer tubes (CPT, BD Biosciences) and centrifuged within 2 hours at 1,600*g* for 15 minutes. Fractions containing peripheral blood mononuclear cells (PBMCs) and plasma were collected as per manufacturer’s recommendations. Samples were centrifuged at 300*g* for 15 minutes to separate plasma from PBMCs. Plasma was collected and stored at –80°C or –20°C until use. PBMCs were washed twice with phosphate-buffered saline (PBS) and centrifuged (300*g*, 15 minutes) as in prior steps. PBMCs were resuspended in PBS, counted using trypan blue, Burker chambers, and microscopy. Following counting PBMCs were centrifuged (300*g*, 15 minutes) as in prior steps and either used immediately in downstream flow cytometry or resuspended in FBS containing 10% DMSO and frozen at –180°C until use. Saliva was collected into clean plastic cups, transferred to either cryovials or 15 mL Falcon tubes using a Pasteur pipette, and stored at –80°C or –20°C until use. To remove insoluble material, saliva samples were centrifuged at 1,000*g* for 10 minutes and the supernatant collected, either prior to freezing or upon thawing before further use. For serum samples, blood was collected into serum Vacutainer tubes (BD Biosciences) and processed according to clinical routine at Örebro University Hospital. Serum was stored at –80°C or –20°C until use.

### Assessment of SARS-CoV-2–specific IgG responses in plasma and saliva.

Prior to serological assessment, plasma and saliva samples were heat inactivated at 56°C for 30 minutes. ELISA to assess vaccine-specific antibodies was performed as previously described (23, 47, 48) with modifications. Half-area high binding ELISA plates (Greiner Bio-One or Corning) were coated at 4°C overnight with 50 ng/well of either SARS-CoV-2 spike protein or soluble RBD (gifts from Neil King, University of Washington, Seattle, Washington, USA) at 1 μg/mL in PBS. ELISA plates were washed 3 times using PBS containing 0.05% Tween-20 (PBS-T); all subsequent washing steps were performed identically unless otherwise specified. Plates were blocked for 1 hour at room temperature (RT) using 5% (w/v) milk powder in PBS. Plasma or saliva samples were diluted 5-fold in PBS with 5% milk. In total, 50 μL serially diluted samples were added to ELISA plates and incubated for 2 hours at RT, followed by a wash step. For assessment of antibody avidity by chaotropic ELISA, parallel plates were treated with either PBS or 1.5M NaSCN, 50 μL/well, for 10 minutes followed by washing. Subsequently, plates were incubated 1 hour at RT with 50 μL/well peroxidase-conjugated anti-human-IgG or anti-IgA secondary antibody (109-035-008 and 109-035-011, respectively; Jackson ImmunoResearch) diluted 1:5,000 (IgG) or 1:2,000 (IgA) diluted in PBS + 5% milk. Plates were then washed. For detection, 50 μL/well 1-step Ultra TMB substrate (Thermo Fisher Scientific) was added and incubated for 5 minutes at RT. Peroxidase activity was stopped using 50 μL/well 1M H_2_SO_4_. Plates were read at 450 nm with 570 nm background correction using a VarioSkan Lux Multimode reader (Thermo Fisher Scientific). For saliva assessments, plates were read at 450 nm with 550 nm background correction using an EnSpire Multilabel Reader (PerkinElmer). Optical density (OD) at 570/550 nm was subtracted from the OD at 450 nm, and the resultant OD was used in all subsequent calculations. A 4-point logarithmic (4PL) nonlinear curve fit was performed using Graphpad Prism version 9.1 or 10. Titers giving half-maximal OD (ED_50_) or endpoint titers, were used as readout for plasma and saliva, respectively. Calibration to the WHO First International Standard NIBSC 20/136 (1000 IU) was calculated as follows: *ED_50_(sample)/ED_50_(standard)* × *1,000 IU*. For assessment of SARS-CoV-2 spike–specific avidity, the avidity index was calculated as the percentage of binding remaining in 1.5M NaSCN-treated plates as compared with untreated plates: *ED_50_(PBS)/ED_50_(NaSCN)* × *100%*.

### Assessment of RBD-specific fraction of spike-binding plasma IgG by competition ELISA.

ELISA plates were coated with 1 μg/mL spike protein, washed, and blocked using the same procedure as described for binding ELISA. Plasma samples were serially diluted in V-bottom plates and incubated with either 20 μg/mL soluble RBD in 5% milk buffer, or buffer alone, for 30 minutes at RT. Following incubation with soluble protein, plasma dilutions were transferred to spike-coated ELISA plates and incubated a further 1.5 hours at RT. Detection and development were carried out as described above for binding ELISA. The RBD binding fraction was calculated as the ratio of binding detected in RBD-competed samples compared with noncompeted: *1.0 – (ED_50_[competed]/ED_50_[noncompeted])*.

### Neutralization assays.

Live virus neutralization assay was performed at Umeå University as previously described (49) with minor modifications. Briefly, SARS-CoV-2 WT strain (SARS-CoV-2/01/human/2020/SWE) were grown on Vero E6 cells. For virus neutralization assay, samples were serially diluted 5-fold in serum-free DMEM supplemented with 0.2% penicillin/streptomycin. Diluted serum samples were incubated with 1,000 PFU/well SARS-CoV-2 virus for 30 minutes at 37°C. Following preincubation, serum-virus mix was added to Vero E6 cells, seeded in Greiner CELLSTAR 96-well plates (Greiner Bio-One) at density of 1 × 10^4^/well, 1 day prior to experiment. Cells were infected for 8 hours and then fixed in 4% formaldehyde for 40 minutes. Plates were washed with PBS, and cells were permeabilized for 10 minutes at RT using 0.5% Triton-X and 20 mM glycine in PBS. Plates were blocked for 30 minutes at RT using 2% BSA in PBS followed by staining of infected cells using a rabbit anti–SARS-CoV-2 nucleocapsid antibody (Sino Biological, 40143-R001) for 1 hour at RT, followed by a goat anti–rabbit IgG (H+L) AF-488 secondary antibody for 30 minutes at RT (Thermo Fisher Scientific, A-11034). Plates were counterstained with DAPI (0.1 μg/mL) for 10 minutes. Fluorescence signal was measured using a TROPHOS plate RUNNER HD instrument (Trophos SA). The half-maximal inhibitory dilution reciprocal (ID_50_) was determined by 4-parameter nonlinear regression performed in Graphpad Prism 9.0.

### Luminex and MSD assays.

SARS-CoV-2 spike antigen was measured in plasma samples using the S-PLEXSARS-CoV-2 spike kit antigen capture ECL immunoassay platform (Meso Scale Discovery [MSD]). Assays were conducted according to manufacturer instructions. Seven-point calibration curve and negative control consisting of assay diluent were run in duplicate on each plate. Plates were read using the MESO QuickPlex SQ 120 instrument. Raw signal was converted to serum concentration based on linear regression to the 7-point calibration curve.

Serum concentrations of cytokines and chemokines were analyzed using a custom Procartaplex human cyto-/chemokine Luminex panel (Invitrogen) according to the manufacturer’s instructions. Luminex data were collected on a MagPix instrument. 4PL curve fits of protein standard curves and interpolation of sample MFI values to plasma concentrations were performed using Belysa Immunoassay Curve fitting software (MilliporeSigma). Values below the lower limit of quantitation (LLOQ) were set to LLOQ for fold change calculations and statistical comparison.

### Innate flow cytometry.

For assessment of innate immune cell populations in blood, up to 5 × 10^6^ freshly isolated PBMCs were surface stained for innate phenotypic markers. Remaining RBCs were removed using BD Pharm Lyse lysis buffer according to manufacturer instructions prior to staining. Cells were stained with Live/Dead Aqua viability dye (Invitrogen) for 5 minutes, followed by addition of FcR blocking reagent (Miltenyi Biotec) for 5 minutes. Two panels were used to characterize cell subsets and activation markers. Panel 1 (Supplemental Figure 13A): CD66 FITC (TET2; Miltenyi Biotec), CD86 (FUN-1;BD Biosciences), HLA-DR PE-Texas Red(TU36; Invitrogen) or HLA-DR PE-Dazzle 594 (L243; BioLegend), CD123 PerCP-Cy5.5 (7G3; BD Biosciences), CCR7 PE-Cy7 (G043H7; BioLegend), CD11c APC (3.9; BioLegend), CD14 APC-Cy7 (M5E2; BioLegend), CD16 BV421 (38G; BioLegend), CD3 BV510 (SP34-2; BD Biosciences), CD19 BV510 (HIB19; BioLegend), CD20 BV510 (2H7; BioLegend), and CD56 BV510 (B159; BD Biosciences). Panel 2 (Supplemental Figure 13B): CD66 FITC (TET2; Miltenyi Biotec), LOX-1 PE (15C4; BD Biosciences), HLA-DR PE-Texas Red(TU36; Invitrogen) or HLA-DR PE-Dazzle 594 (L243; BioLegend), CD11b PerCP-Cy5.5 (ICRF44; BioLegend), CCR7 PE-Cy7 (G043H7; BioLegend), CD14 APC (M5E2; BioLegend), CD3 APC-Cy7 (SP34-2; BD Biosciences), CD19 APC-Cy7 (HIB19; BioLegend), CD20 APC-Cy7 (L27; BioLegend), CD56 BV510 (HCD56; BioLegend), and CD16 BV421 (38G; BioLegend). Samples were surface stained for 20 minutes at 4°C, washed with PBS, fixed with 1% formaldehyde, and acquired on a Gallios flow cytometer (Beckman Coulter). Data were analyzed using FlowJo version 10 (FlowJo Inc.).

### Assessment of SARS-CoV-2 specific memory B cell responses.

Fluorescently conjugated antigen probes were prepared as previously described (23, 48). Briefly, SARS-CoV-2 spike and soluble RBD proteins were biotinylated using Sulfo-NHS-biotin (Thermo Fisher Scientific) according to manufacturer instructions. Excess biotin was removed using Slide-A-Lyzer mini dialysis devices (Thermo Fisher Scientific). Prior to staining of PBMCs, fluorescent spike and RBD tetramers were constructed by stepwise incubation of biotinylated spike and RBD proteins with either Streptavidin-APC, Streptavidin-PE, or Streptavidin-BV421 (BioLegend) on ice, for a 4:1 final molar ratio of protein to streptavidin conjugate. Frozen PBMCs were thawed in a 37°C water bath and transferred to RPMI complete medium (HyClone) supplemented with 10% FBS (Thermo Fisher Scientific), 1% penicillin/streptomycin, and 1% L-glutamine (R10). Cells were washed twice with R10, rested for 1–3 hour (37°C, 5% CO_2_), and counted using Trypan blue and an automated cell counter. One million to 3 million PBMCs were transferred to FACS tubes, washed with cold PBS containing 2% FBS, and stained with 100 ng of spike-PE tetramer, spike-APC tetramer, and RBD-BV421 tetramer for 20 minutes at 4°C. Cells were then surface stained with anti–human IgM-PerCP-Cy5.5 (G20-127; BD Biosciences), CD3-BV510 (SP34-2; BD Biosciences), CD123-BV510 (6H6; BioLegend), CD19-ECD (J3-119; Beckman Coulter), CD16-BV510 (3G8 BD Biosciences), HLA-DR- BV650 (L243; BioLegend), IgG-BV786 (G18-145; BD Bioscience), CD20-BV605 (2H7; BioLegend), CD14-BV510 (M5E2; BioLegend), IgD-FITC (Polyclonal; Southern Biotech), CD27-PE-Cy7 (M-T271; BioLegend), and CD38-APC-Cy7 (HIT2; BioLegend) and 7AAD viability dye (Invitrogen) for 20 minutes at 4°C. Samples were washed with cold PBS with 2% FBS, fixed using 1% paraformaldehyde (PFA), and acquired on an LSRFortessa flow cytometer (BD Biosciences). A small subset of samples were acquired using a FACSAria III Fusion instrument due to technical constraints. Data were analyzed using FlowJo version 10 (FlowJo Inc.). Representative gating is shown in Supplemental Figure 14A.

### Assessment of SARS-CoV-2 specific memory T cell responses.

Antigen-specific T cells were assessed by peptide stimulation followed by intracellular cytokine staining (ICS) as previously described (23). Briefly, PBMCs were thawed as described for memory B cell assessment. One million to 2 million PBMCs were stimulated overnight at 37°C, 5% CO_2_, with either 2 μg/mL overlapping peptides (15-mers overlapping by 11) covering the SARS-CoV-2 spike protein (JPT), 1 μg/mL Staphylococcal Enterotoxin B (Sigma-Aldrich) as positive control, or R10 with 0.8% DMSO as negative control. Brefeldin A (Thermo Fisher Scientific) was added to all conditions for a final concentration of 10 μg/mL. Following stimulation, cells were washed twice with PBS, stained first with Live/Dead Fixable Blue or Live/Dead Fixable Aqua viability dyes (Invitrogen) for 5 minutes at 4°C followed by anti–human CCR7-BV421 (G043H7; BioLegend), CD8a-BV711 (RPA-T8, BioLegend), CD4-PE-Cy55 (S3.5; Invitrogen), and CD45RA-BV650 (5H9; BioLegend) for 20 minutes at 4°C. Following surface staining, cells were washed twice with PBS and permeabilized using BD Cytofix/Cytoperm kit (BD Biosciences) for 20 minutes at RT, washed twice, and stained intracellularly with anti–human IL-21-AF647 (3A3-N2.1; BD Biosciences), IL-13-PE (JES10-5A2;BD Biosciences), IL-2-BV605 (MQ1-17H12; BD Biosciences), IL-17A-BV785 (BL168; BioLegend), CD69-ECD (TP1.55.3; Beckman Coulter), CD3-APC-Cy7 (SP34.2; BD Biosciences), IFN-γ–AF700 (B27; BioLegend), and TNF-α–AF488 (Mab11; BD Biosciences) for 20 minutes at 4°C. Following ICS, cells were washed with BD 1× Fix/Perm buffer, resuspended in 1% PFA, and acquired on an LSRFortessa flow cytometer (BD Biosciences). Data were analyzed using FlowJo version 10 (FlowJo Inc.). Representative gatings are shown in Supplemental Figure 2F, Supplemental Figure 3E, and Supplemental Figure 14B. The value for the unstimulated control was subtracted from the peptide stimulated condition, and the result was used as final readout.

### In vitro stimulations and cytokine analysis.

Frozen PBMCs from selected study participants (collected at study baseline and 4 weeks after prime) were thawed at 37°C in a water bath, washed using R10 complete media, and cultured for 24 hours in the presence of either 0.1 μg/mL LPS (Sigma-Aldrich), 2 μg/mL R848 (Invivogen), or 1 μg/mL BNT162b2 vaccine. BNT162b2 used in in vitro experiments consisted of doses planned for discarding, obtained from a routine SARS-CoV-2 vaccination center located at Karolinska University Hospital Solna. Vaccine was collected on the same day of resuspension and stored frozen at –80°C in RNase-free tubes until use in in vitro cultures. Following 24 hours of culture, PBMCs were collected and supernatants were analyzed for cytokine production by proximity extension assay using the OLINK 96 Inflammation panel (Olink Target 96 Inflammation, v.3024) according to manufacturer instructions. Fold changes in normalized protein expression (NPX) were calculated based on unstimulated controls.

### RNA-Seq.

For bulk RNA-Seq analysis, whole blood was collected into PAXgene Blood RNA tubes (PreAnalytiX, BD Biosciences) according to manufacturer instructions and stored at –20°C until sequencing. For library preparation, tubes were thawed overnight at RT, and RNA was extracted using the PAXGene Blood RNA kit (PreAnalytiX, Qiagen) according to manufacturer instructions. Total RNA concentration was measured using a NanoDrop ND-1000 spectrophotometer (Thermo Fisher Scientific). RNA integrity was assessed using the Agilent RNA ScreenTape assay and Agilent 2200 TapeStation (Agilent Technologies) according to manufacturer instructions. In preparation for Illumina sequencing, isolation of mRNA, cDNA synthesis, anchor ligation, amplification, and library indexing were performed using the Illumina Stranded mRNA Prep kit according to manufacturer instructions. Library yield was quantified by Qubit fluorometer (Thermo Fisher Scientific), and quality was assessed by Agilent TapeStation. Indexed DNA libraries were normalized, pooled, and sequenced using a NovaSeq 6000 instrument, S4 flowcell, 2 × 150 base pairs, in paired-end mode.

### Bioinformatic analysis of RNA-Seq data.

Raw reads were preprocessed using the nf-core/rnaseq pipeline (50) version 3.8. Alignment was performed using STAR (51) to the human genome (GRCh38) and gene quantification using Salmon (52). Subsequent processing was performed using R statistical software. Data were filtered to remove reads with log_2_(counts/million) < 1. DEGs were inferred using DESeq2 (53), controlling for participant sex and age as well as experiment batch, and normalizing by median of ratios. Further analysis with DEGs included only significant genes corrected for multiple comparisons with log_2_(fold change) greater than 1. Jaccard similarity index between DEG lists was calculated using GeneOverlap (54). Time points with fewer than 30 DEGs were removed to reduce biased comparisons prior to calculating the Jaccard indexes. The overrepresentation analysis was carried out for this set of DEGs using the KEGG Pathway database (https://www.genome.jp/kegg/pathway.html) (Figure 3A) and the MSigDB subset for Transcriptional Factors Targets (https://www.gsea-msigdb.org/gsea/msigdb/collections.jsp) (Figure 3B). The HUGO Gene Nomenclature Committee (HGNC; https://www.genenames.org/) database (55) was used to retrieve standardized gene lists of interest. The lists of genes were retrieved filtering for immune-associated groups of receptor ligands and receptors, which included the groups for chemokines, IFNs, ILs, TNF, CXCR, CCR, XCR, and CX3CR (total of 157 genes). DEGs presented in Figure 4A were filtered based on this merged gene list. The GSEA was done using the fold change for ranking, and the gene sets were from a previously described blood transcription module (36). GSEA and overrepresentation analysis were performed using clusterProfiler (36, 56). Sequences used for detection and alignment of mRNA vaccine transcripts found in bulk blood RNA-Seq data were sourced from the GitHub repository by Jeong et al. (39). Plots were generated using packages ggplot2 and heatmap. All available scripts and the needed environment used for this analysis are publicly available (github.com/rodrigarc/orebro_study; commit ID: 6e0af0b).

### Statistics.

Statistical comparisons, correlations, and ELISA curve fits were performed using GraphPad Prism 10 for Mac (GraphPad Software) or using R statistical software, unless otherwise specified. Unless otherwise specified in figure legends, study group comparisons of longitudinal data across multiple time points were performed using multiple Mann-Whitney *U* tests, with *P* value correction using the Holm-Šidák method and α threshold 0.05. Paired intragroup comparisons between time points were performed using either Graphpad Prism’s mixed model or Friedman test with Dunn’s multiple comparisons post hoc. Where Friedman test was used, only complete cases were analyzed. Single comparisons between groups at individual time points were performed by Mann-Whitney *U* test. Correlations were performed in Graphpad Prism or R, using nonparametric Spearman or Pearson correlations where appropriate. Hierarchical clustering analysis shown in Figure 3A and Figure 4B was performed in R statistical software using Ward’s method. Statistical tests are further specified in figure legends.

### Study approval.

The study was conducted according to Good Clinical Practice (ICH-GCP) and approved by the Swedish Medical Product Agency (Dnr 5.1-2021-15494, Eudra-CT 2021-000683-30) and the Swedish Ethical Review Authority (Dnr 2021-00055). The study was registered at clinicaltrialsregister.eu (2021-000683-30). All participants signed a written informed consent.

### Data availability.

Data are available upon reasonable request to corresponding author. European Data Regulations preclude open deposition of sensitive personal data into public repositories. A metadata record describing the existing data sets can be found at SciLifeLab Figshare: https://doi.org/10.17044/scilifelab.24941913 Values for all data points in graphs, excluding raw values for RNA-Seq data, are reported in the Supporting Data Values file.

## Author contributions

FH, AR, MNEF, JN, CA, SC, and K Loré designed the clinical study. FH, AR, RAC, MNEF, SC, and K Loré designed experiments. FH, AR, K Lenart, SO, SK, and AMD acquired and processed samples. FH, AR, RAC, K Lenart, SO, YDG, GJ, SK, and AMD generated data. FH, AR, RAC, YDG, GJ, ME, JN, CA, MNEF, SJ, and K Loré analyzed and interpreted data. All authors critically revised the manuscript. FH, RAC, GJ, and K Loré performed statistical analysis. FH and AR contributed equally to the study.

## Supplementary Material

Supplemental data

Supporting data values

## Figures and Tables

**Table 1:**
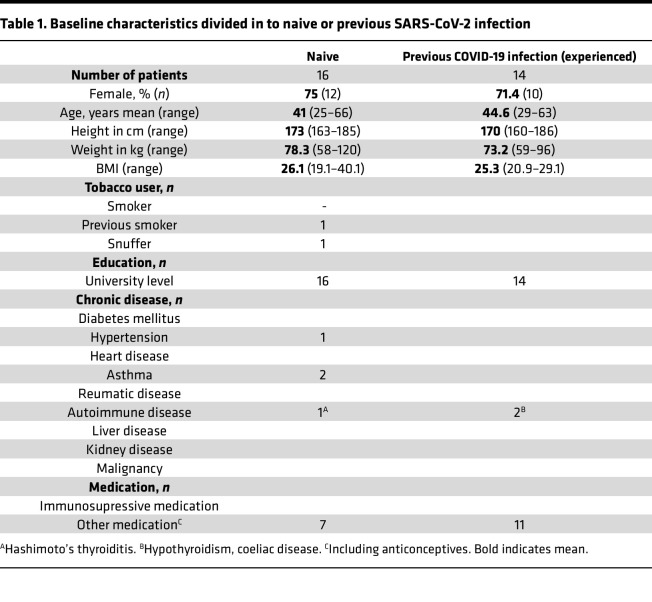
Baseline characteristics divided in to naive or previous SARS-CoV-2 infection

**Table 2:**
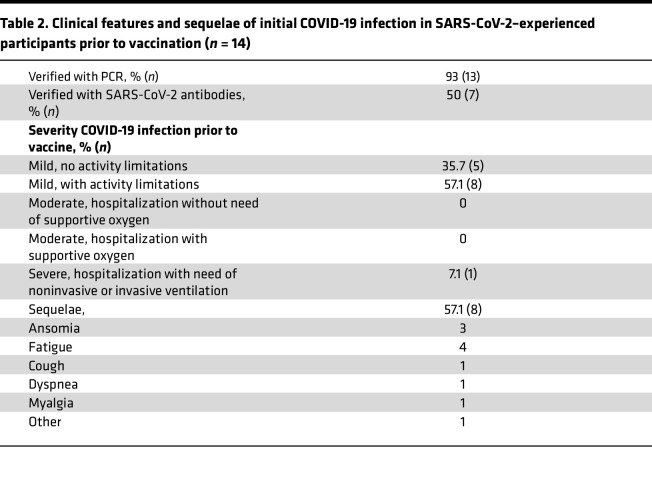
Clinical features and sequelae of initial COVID-19 infection in SARS-CoV-2–experienced participants prior to vaccination (*n* = 14)

## Version 1 05/08/2024

Electronic publication
