# Supplementary material for: Modulation of innate immune response to mRNA vaccination after SARS-CoV-2 infection or sequential vaccination in humans
Source: JCI Insight. 2024 May 8;9(9):e175401. doi: 10.1172/jci.insight.175401 (PMC11141904; doi:10.1172/jci.insight.175401)
Supplement: Supplemental data [file jciinsight-9-175401-s274.pdf]

Modulation of innate immune activity after

infection or sequential mRNA vaccination in humans

Hellgren F[1,2]*, Rosdahl A[3]*, Arcoverde Cerveira R[1,2], Lenart K[1,2], Ols S[1,2], Gwon Y-D[5], Joas G[1], Kurt S[4], Delis A-M[4], Evander M[5], Normark J[5], Ahlm C[5], Forsell M[5], Cajander S[3,6], Loré K[1,2]

[1]Division of Immunology and Allergy, Department of Medicine Solna, Karolinska Institutet, Stockholm, Sweden & Karolinska University Hospital, Stockholm, Sweden. [2]Center for Molecular Medicine, Karolinska Institutet, Stockholm, Sweden. [3]Department of Infectious Diseases, Örebro University Hospital, Örebro, Sweden. [4]Clinical Epidemiology and Biostatistics Unit, Örebro University Hospital, Sweden. [5]Department of Clinical Microbiology, Umeå University, Umeå, Sweden. [6]School of Medical Sciences, Faculty of Medicine and Health, Örebro University, Örebro Sweden.

**equal contribution*

Corresponding author: Karin Loré (Karin.lore@ki.se)

Supplementary Figures

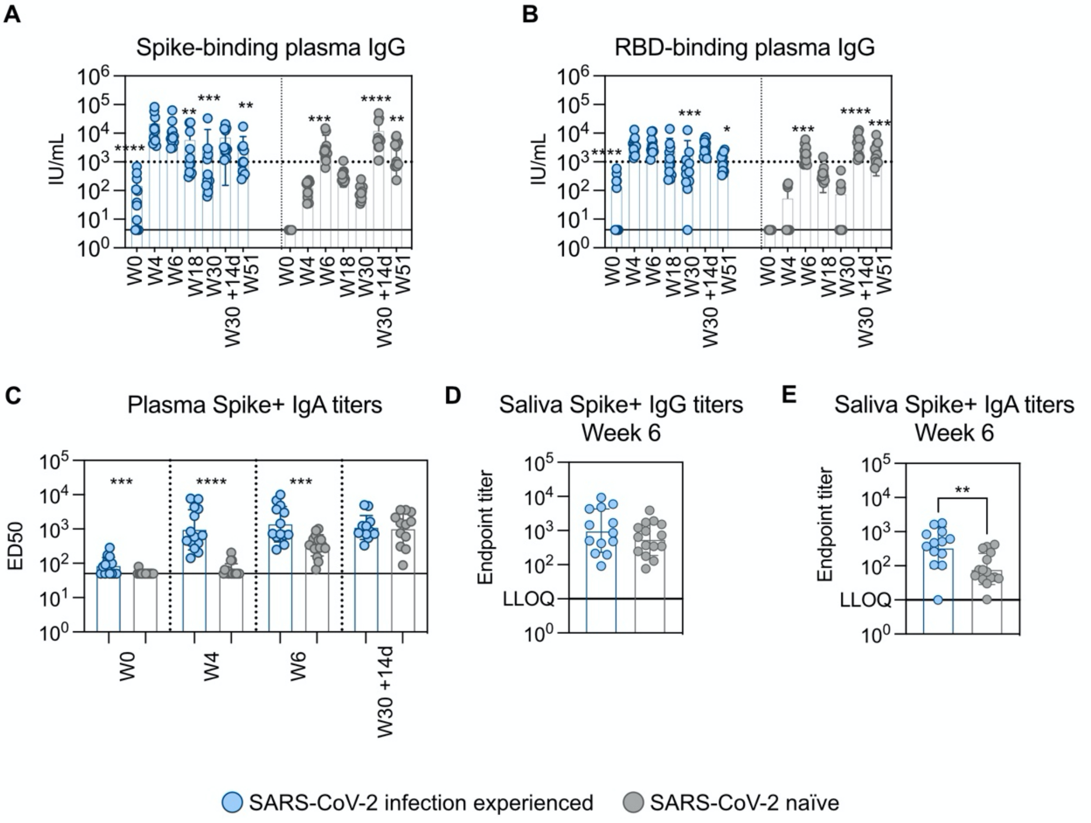

Figure S1

Figure S1: Plasma and saliva Spike- and RBD-binding antibody titers. A-B: Longitudinal comparisons of plasma IgG binding full spike protein **(A)** or RBD **(B).** Comparison performed using Friedman test with Dunn's multiple comparisons post hoc, comparing each timepoint to W4. N = 23 (complete cases only). **C:** Plasma Spike-binding IgA titers, data shown as ED50 values. Groups were compared by multiple Mann-Whitney test with comparison between groups at each timepoint and p value adjustment using the Holm-Sidak method (alpha threshold 0.05). Number of study participants shown per timepoint: Week 0 = 30; Week 4 = 30; Week 6 = 29; Week 30+14 days = 24. **D-E**: Week 6 saliva Spike-binding IgG (D) and IgA (E) levels measured by ELISA, data shown as endpoint titers. Group comparison by Mann-Whitney test. Bar and error indicate geometric mean +/- geometric SD. N = 28.

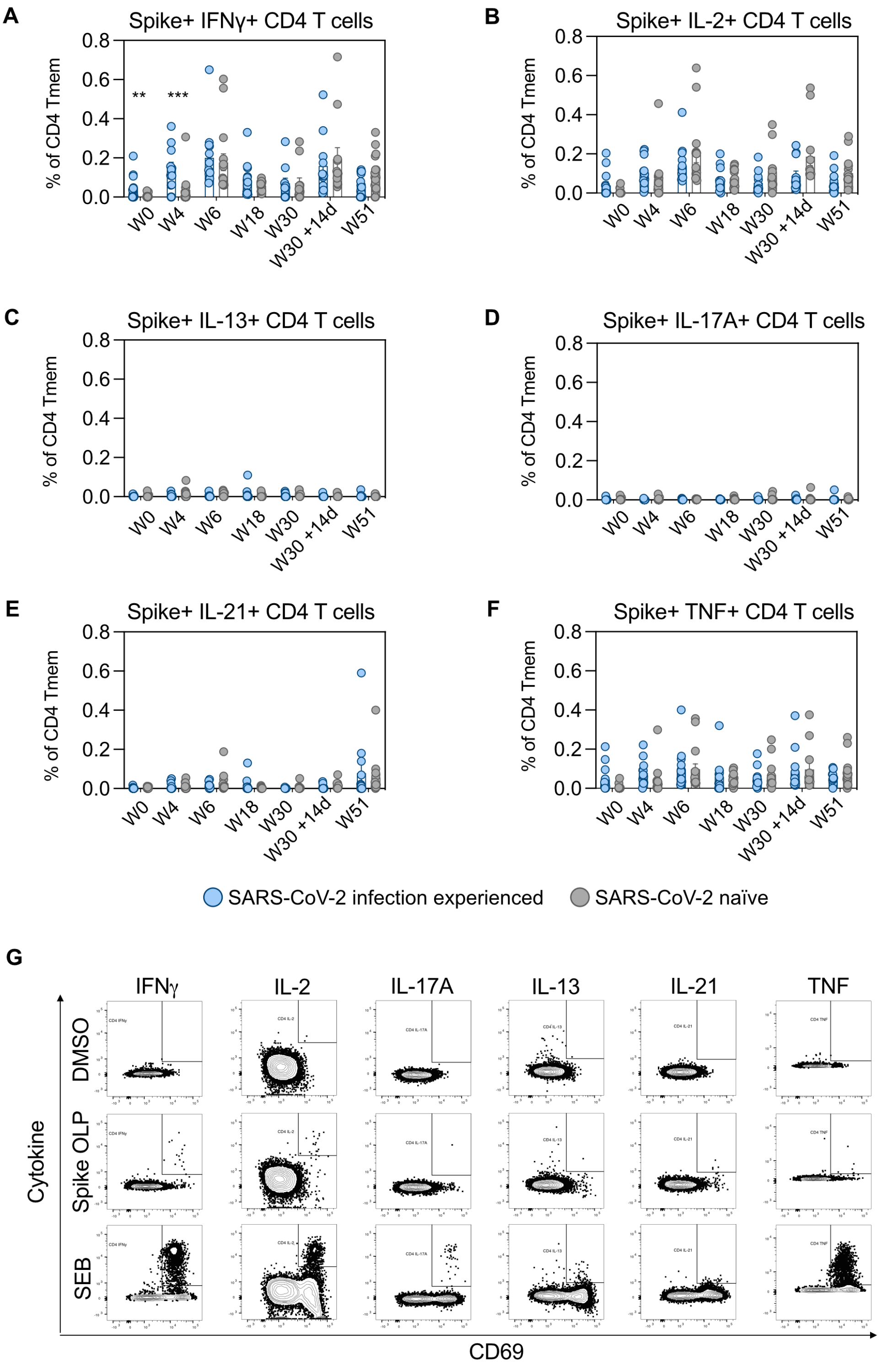

Figure S2

Figure S2: Assessment of cytokine-producing CD4 T cells in response to SARS-CoV-2 spike overlapping peptide stimulation. A-F: Spike-specific CD4 T cells producing IFNy **(A)**, IL-2 **(B),** IL-13 **(C),** IL-17A **(D),** IL-21 **(E)** or TNF **(F)** in response to SARS-CoV-2 Spike overlapping peptide stimulation. Data shown as percentage of CD4 memory T cells. Number of participants shown per timepoint: Week 0 = 30; Week 4 = 28; Week 6 = 29; Week 18 = 28; Week 30 = 23; Week 30 + 14d =

24; Week 51 = 28. Groups were compared using multiple Mann-Whitney test with p value adjustment for multiple comparisons using the Holm-Sidak method, alpha threshold 0.05. **G:** Representative gating of cytokine-producing/CD69+ memory T cells. Line and error bars indicate mean +/- SEM. Gating strategy for the identification of memory T cell population shown in figure S14B.

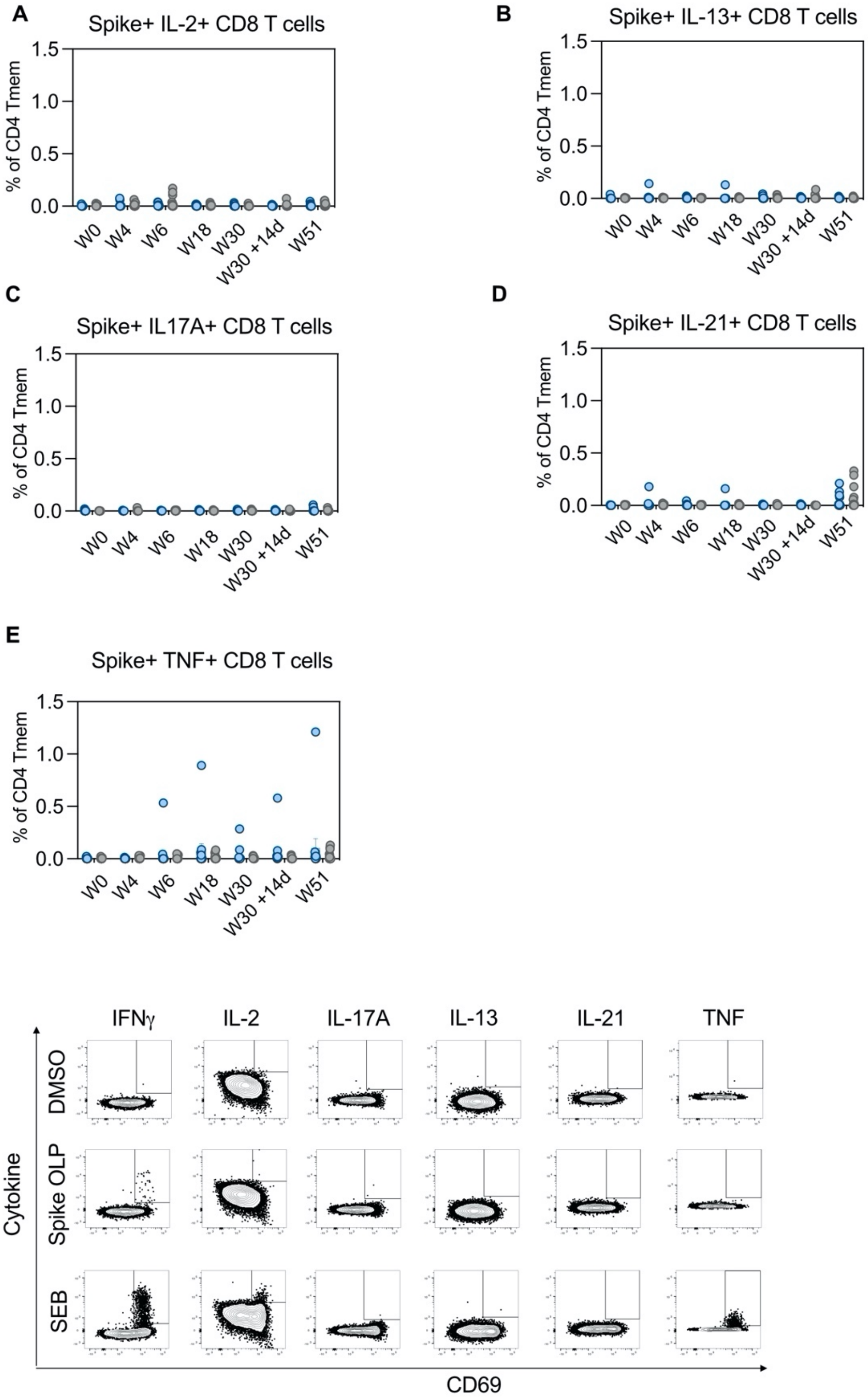

Figure S3

Figure S3: Assessment of cytokine-producing CD8 T cells in response to SARS-CoV-2 spike overlapping peptide stimulation. A-F: Spike-specific CD4 T cells producing IL-2 **(A),** IL-13 **(B),** IL-17A **(C),** IL-21 **(D)** or TNF **(E)** in response to SARS-CoV-2 Spike overlapping peptide stimulation. Data shown as percentage of CD4 memory T cells. Groups were compared using multiple Mann-Whitney test with p value adjustment for multiple comparisons using the Holm-Sidak method,

alpha threshold 0.05. Number of participants analyzed per timepoint: Week 0 = 30; Week 4 = 28; Week 6 = 29; Week 18 = 28; Week 30 = 23; Week 30 + 14d = 24; Week 51 = 28. **F:** Representative gating of cytokine-producing/CD69+ memory T cells. Line and error bars indicate mean +/- SEM. Gating strategy for the identification of memory T cell population shown in figure S14B.

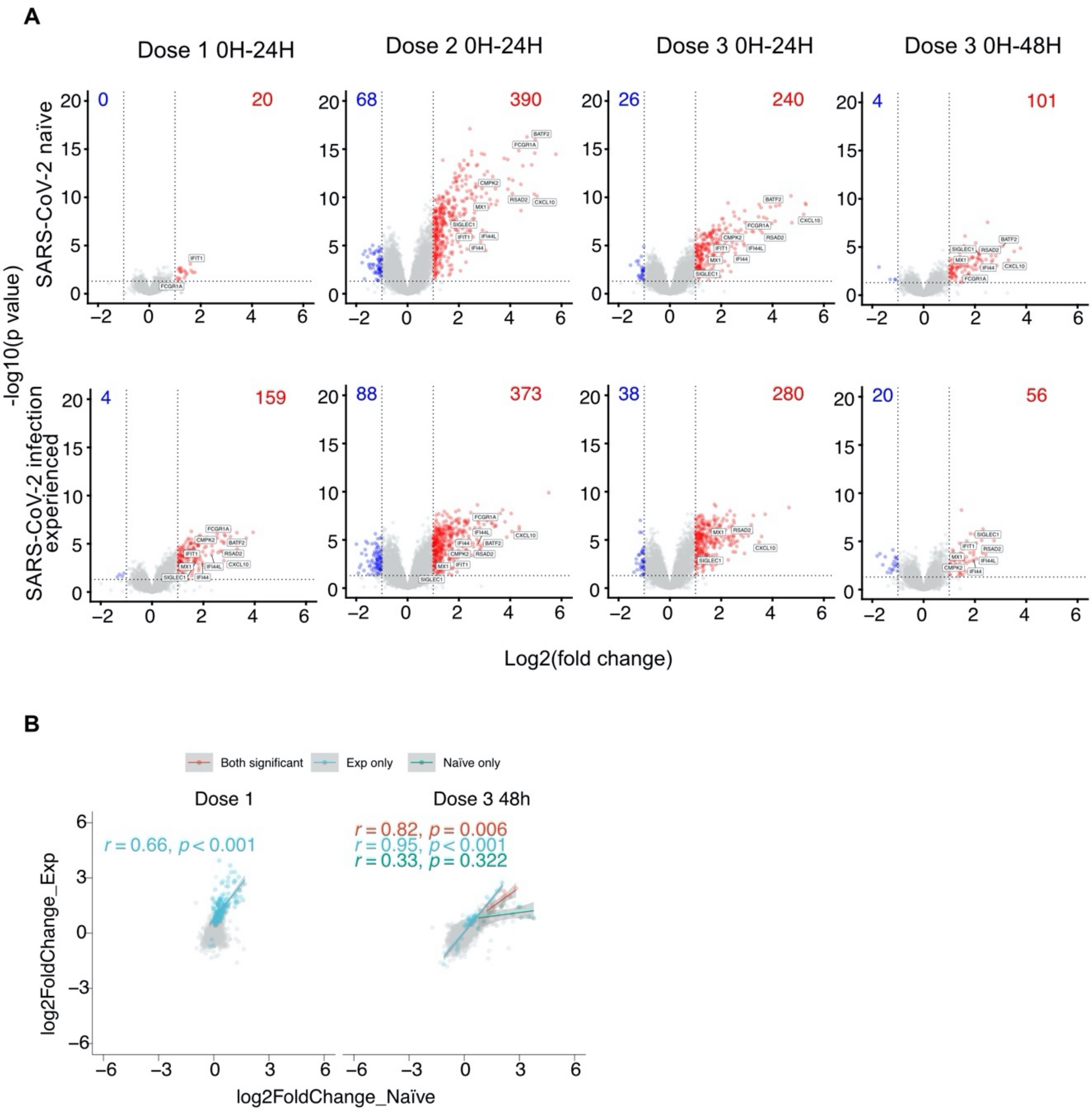

Figure S4

Figure S4: Assessment of differentially expressed genes in response to mRNA vaccination. A: Volcano plots displaying differentially regulated genes in peripheral blood measured by RNASeq, unadjusted p value. Fold changes and p values for each sample group generated by paired t-test between pre-vaccination (0H) and post-vaccination samples (24h or 48h) at each vaccine dose. Total number of differentially up- or down-regulated genes are indicated in each plot. Cut-off for significant differential regulation were log2(fold change) > 1 , unadjusted p value < 0.05. **B:** Pearson's correlation of fold changes in individual genes identified as significantly differentially regulated in any group, Dose 1 and Dose 3. Cut-off for significant differential regulation were log2(fold change) > 1 , FDR-adjusted p value < 0.05. N = 15.

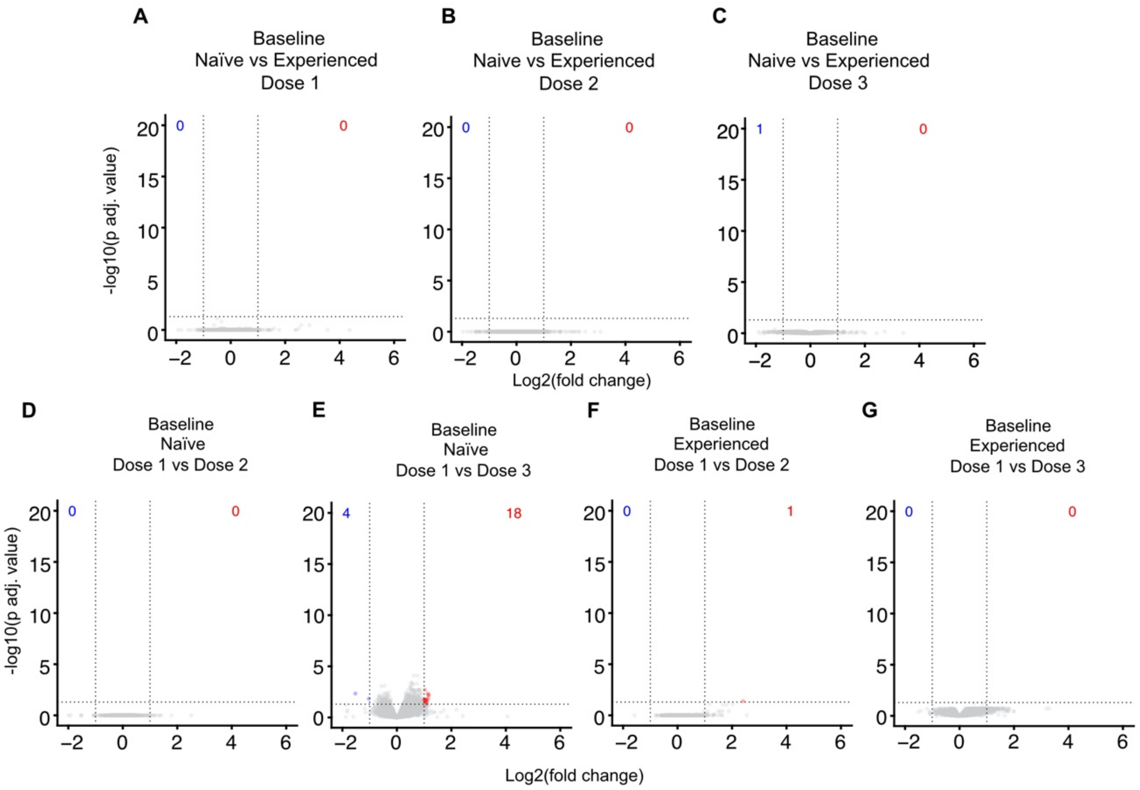

Figure S5

Figure S5: Assessment of differentially expressed genes at baseline between doses. A-C: Volcano plots displaying differentially regulated genes in peripheral blood measured by RNASeq. Fold changes and p values for each sample group generated by unpaired t-test between SARS-CoV2 infection experienced and infection naïve study groups at the baseline timepoint (day of vaccination) for each vaccine dose. Total number of differentially up- or down-regulated genes are indicated in each plot. Cut-off for significant differential regulation were log2(fold change) > 1 , adjusted p value < 0.05. **D-G:** Volcano plots displaying differentially regulated genes in peripheral blood measured by RNASeq. Fold changes and p values for each sample group were generated by paired t-test between day 0 samples at first dose (study start), and day 0 samples for subsequent vaccine doses to detect any potential lasting transcriptomic changes. Total number of differentially up- or down-regulated genes are indicated in each plot. Cut-off for significant differential regulation were log2(fold change) > 1 , adjusted p value < 0.05. N = 15.

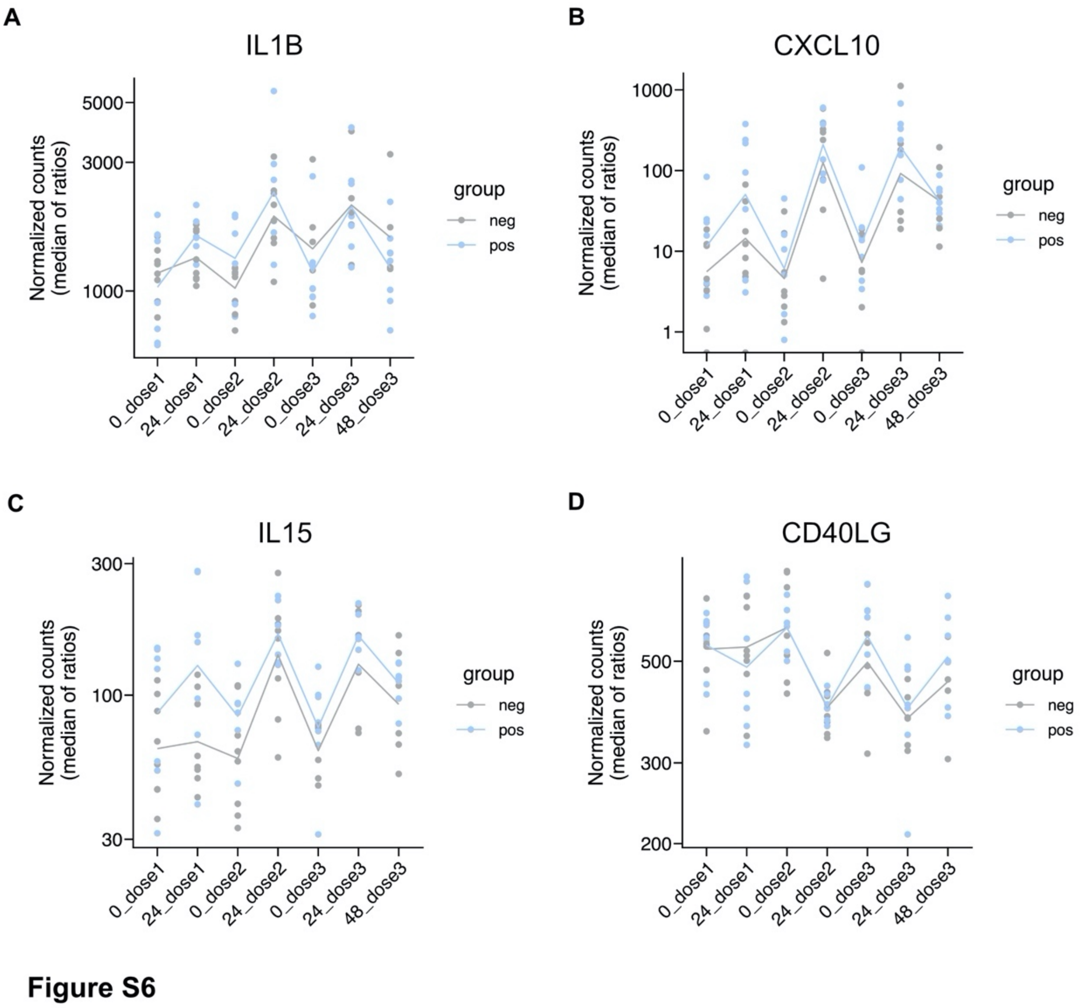

Figure S6: Detection of selected cytokines before and after mRNA vaccination in whole blood transcriptomic data. A-D: Transcript levels of IL1B **(A),** CXCL10 **(B),** IL15 **(C)**, **(D)**, and CD40LG **E)** before and after vaccination. N = 15.

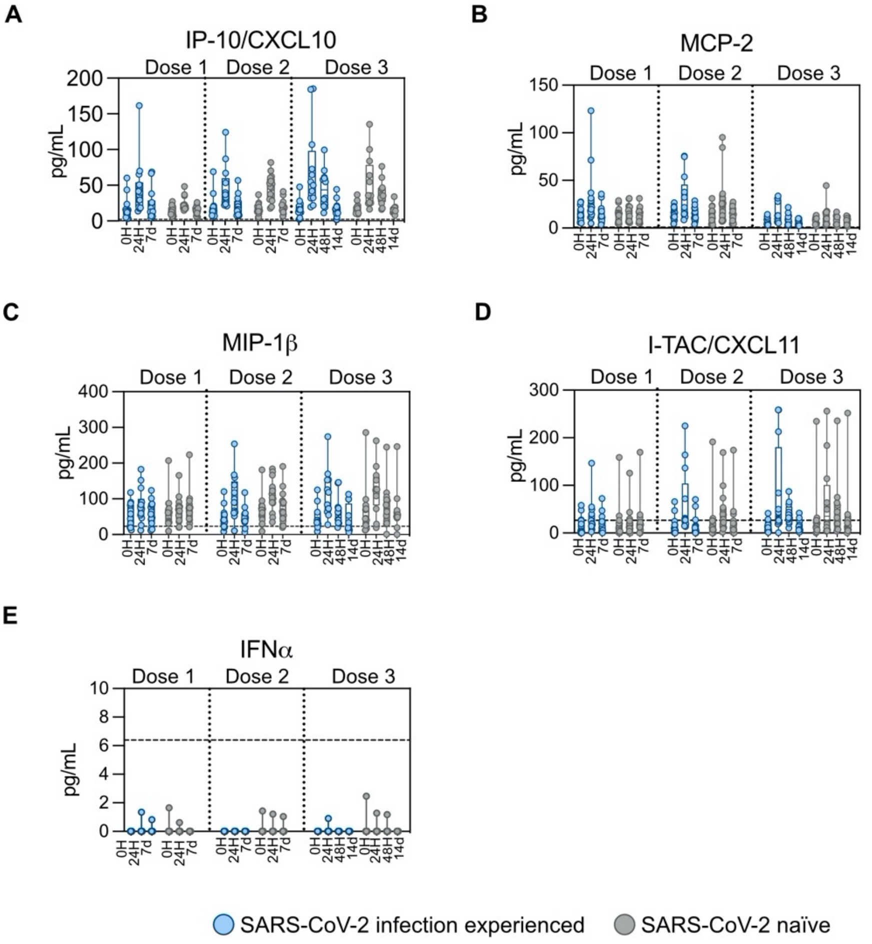

Figure S7

Figure S7: Serum levels of selected cytokines before and after mRNA vaccination. A-E: Serum levels of CXCL10 **(A),** MCP-2 **(B),** MIP-1 beta **(C)**, CXCL11 **(D)**, and Interferon alpha **(E)** before and after vaccination, measured by Luminex. Box-and-whiskers indicating min-max. Number of study participants shown (all panels): Dose 1 = 30. Dose 2 = 29. Dose 3 = 24. Groups were compared by multiple Mann-Whitney test with comparison between groups at each timepoint and p value adjustment using the Holm-Sidak method (alpha threshold 0.05). Dashed line indicates lower limit of quantitation (highest limit of any plate in analysis was used).

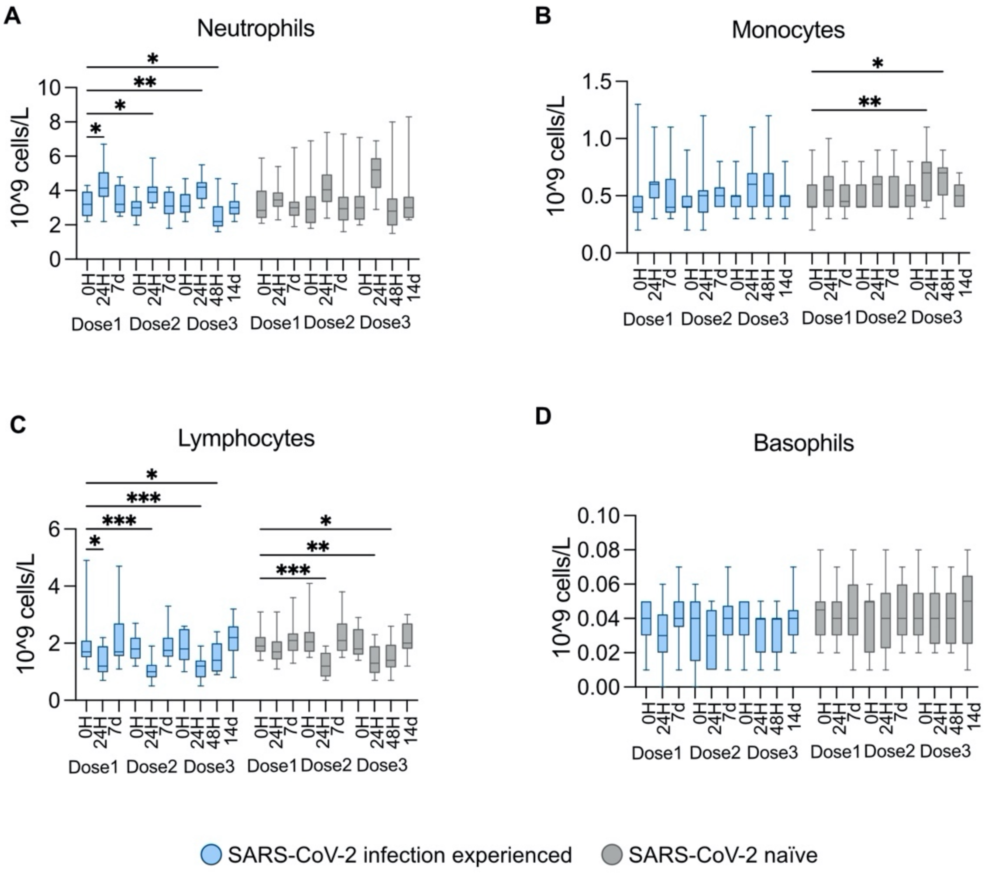

Figure S8

Figure S8: Absolute counts of immune populations in peripheral blood before and after mRNA vaccination. A-D: Absolute counts of neutrophils **(A)**, monocytes **(B),** lymphocytes **(C)** and basophils **(D)** measured by complete blood count assay according to clinical routine at Örebro University Hospital. Box-and-whiskers indicating min-max. Number of participants analyzed: Dose 1 = 30; Dose 2 = 29; Dose 3 = 24. Within-group comparisons across timepoints performed using Prism 10 mixed-effects model with Dunn's multiple comparisons post hoc, comparing each timepoint to study start (Dose 1 0H).

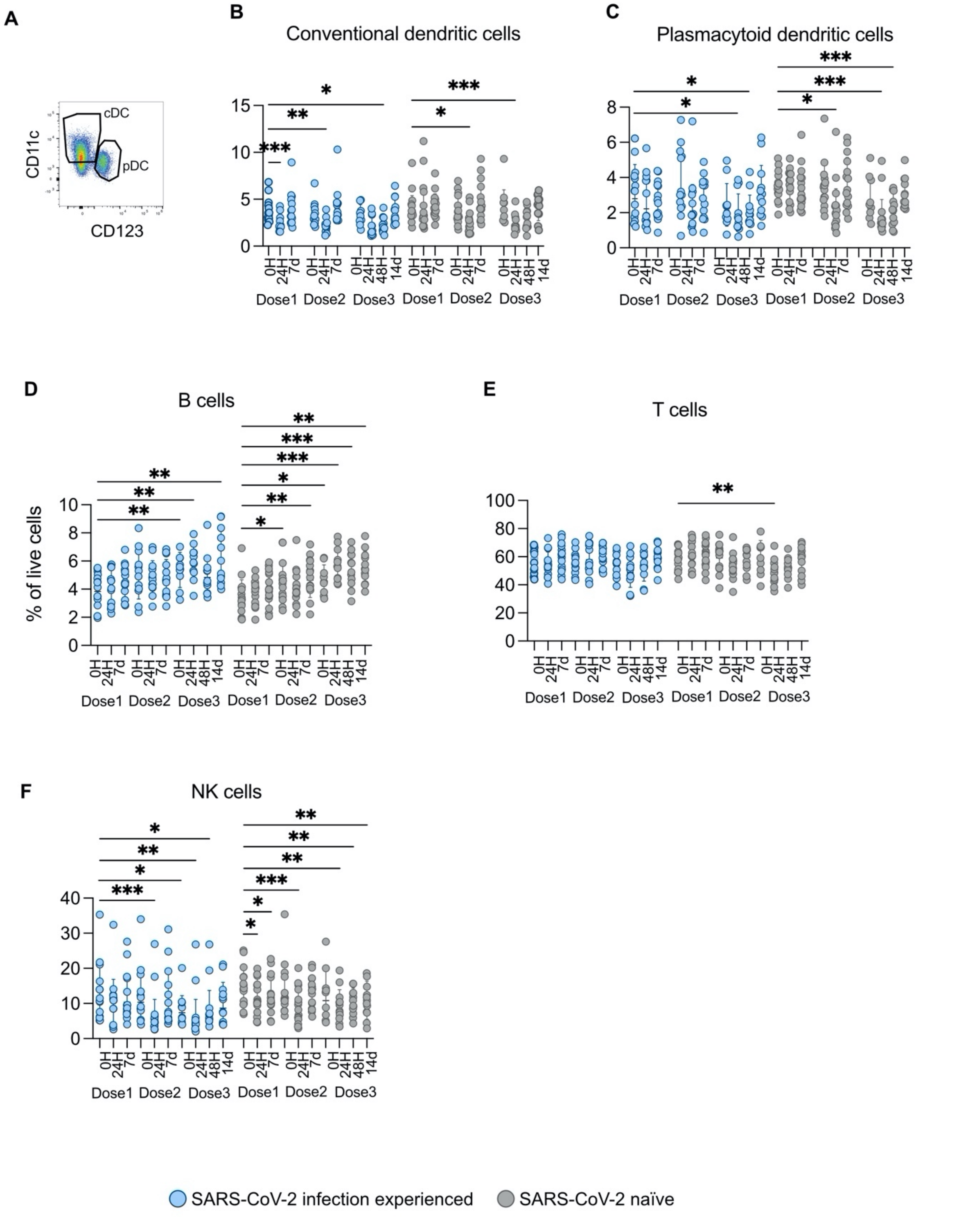

Figure S9

Figure S9: A: Representative gating of conventional dendritic cells and plasmacytoid dendritic cells. **B-C:** Quantification of conventional dendritic cells (cDC) **(B)** and plasmacytoid dendritic cells (pDC) **(C)** as a proportions of total gated HLA-DR+ cells. **D-F:** Quantification of B cells **(D)**, T cells **(E)** and NK cells **(F)** as proportions of total gated live single cells. Number of participants analyzed: Dose 1 = 29. Dose 2 = 28 (0H = 27) Dose 3 = 24 (0H = 19). Within-group comparisons across timepoints

performed using Prism 10 mixed-effects model with Dunn’s multiple comparisons post hoc, comparing each timepoint to study start (Dose 1 0H).

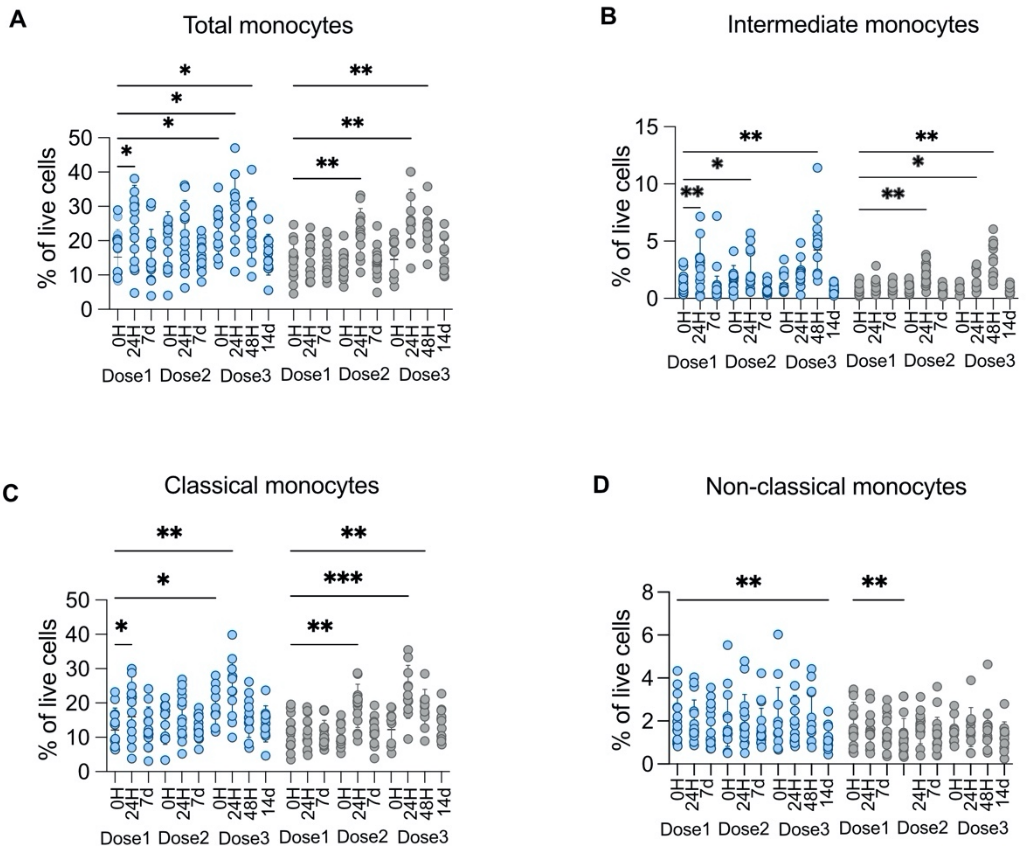

Figure S10

Figure S10: Longitudinal comparisons of monocyte populations in blood measured by flow cytometry. A-D: Longitudinal comparison of total monocytes **(A),** intermediate monocytes **(B),** classical monocytes **(C)** and non-classical monocytes **(D).** Within-group comparisons across timepoints performed using Prism 10 mixed-effects model with Dunn’s multiple comparisons post hoc, comparing each timepoint to study start (Dose 1 0H). Number of participants analyzed: Dose 1 = 29. Dose 2 = 28 (0H = 27) Dose 3 = 24 (0H = 19).

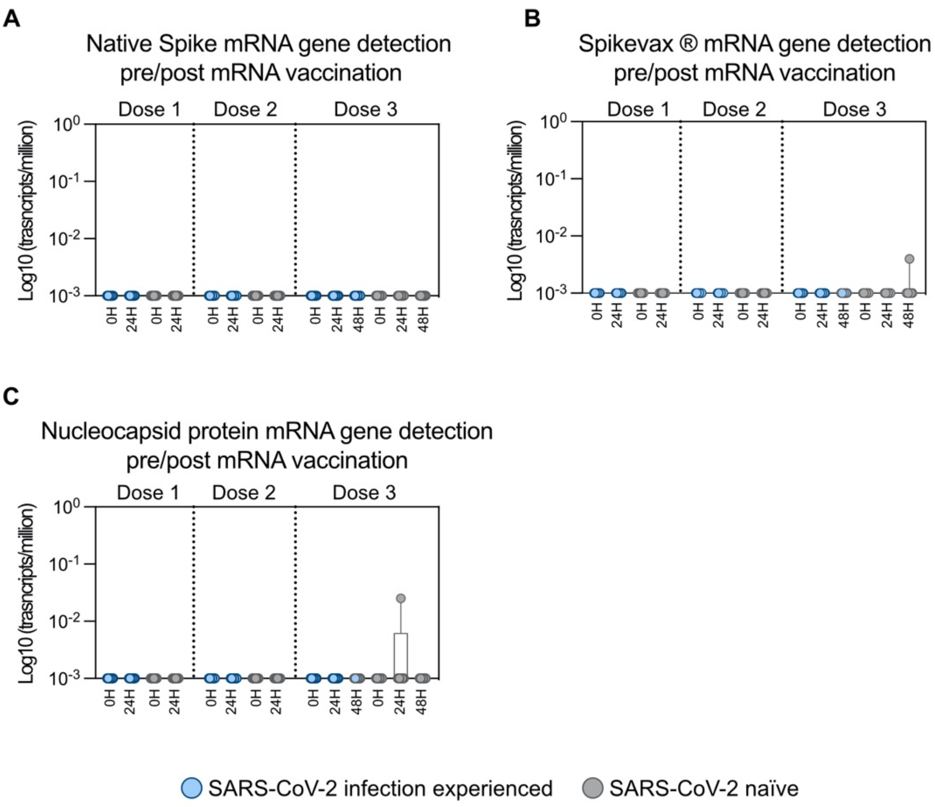

Figure S11

Figure S11: Detection of spike- and nucleocapsid genes in bulk blood RNASeq data across timepoints. Detection of mRNA sequences for native spike protein (A), Spikevax ® mRNA vaccine (B), and virus nucleocapsid protein (C). Box and whiskers indicate min-max. Sequences corresponding to vaccine mRNA were obtained from Jeong et al (59). Sequences used for native spike and nucleocapsid proteins were extracted from the NCBI reference sequence for the SARS- CoV-2 genome: NC_045512.2. N = 15.

A

	Experienced	Naïve
BT before 12mo	3	8
No BT before 12mo	11	7
Lost to follow-up	0	1

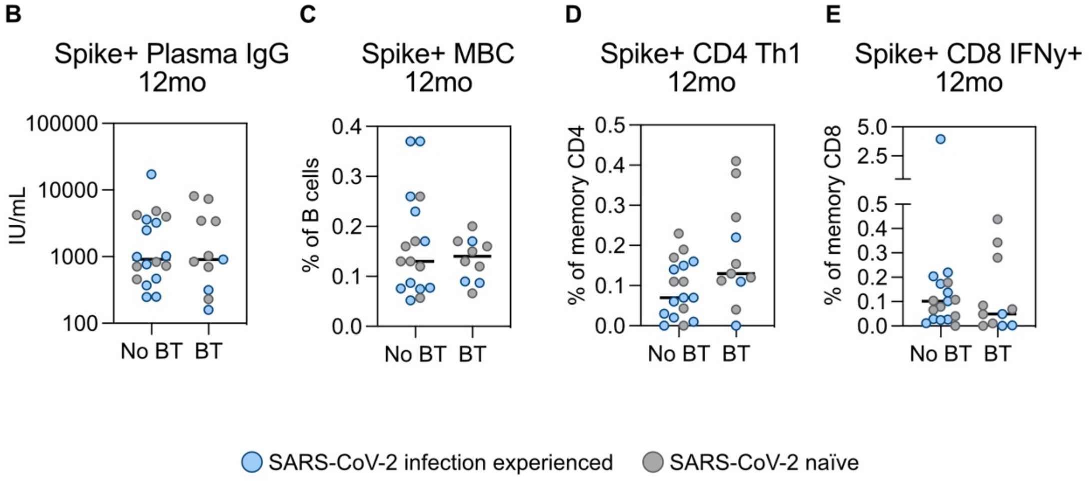

Figure S12

Figure S12: Breakthrough infections and effect on adaptive responses. A: Summary of self-reported breakthrough infections occurring during study follow-up, prior to the 1-year follow up timepoint. **B-D:** SARS-CoV-2 spike-specific binding IgG titers in plasma **(B)**, memory B cells in blood **(C)**, IFNy/IL-2 producing CD4+ memory T cells **(D)** and IFNy-producing CD8 memory T cells **(E)** at time of 1-year follow-up, plotted by breakthrough infection status. N = 28 **(B-E)**. Groups were compared by Mann-Whitney test.

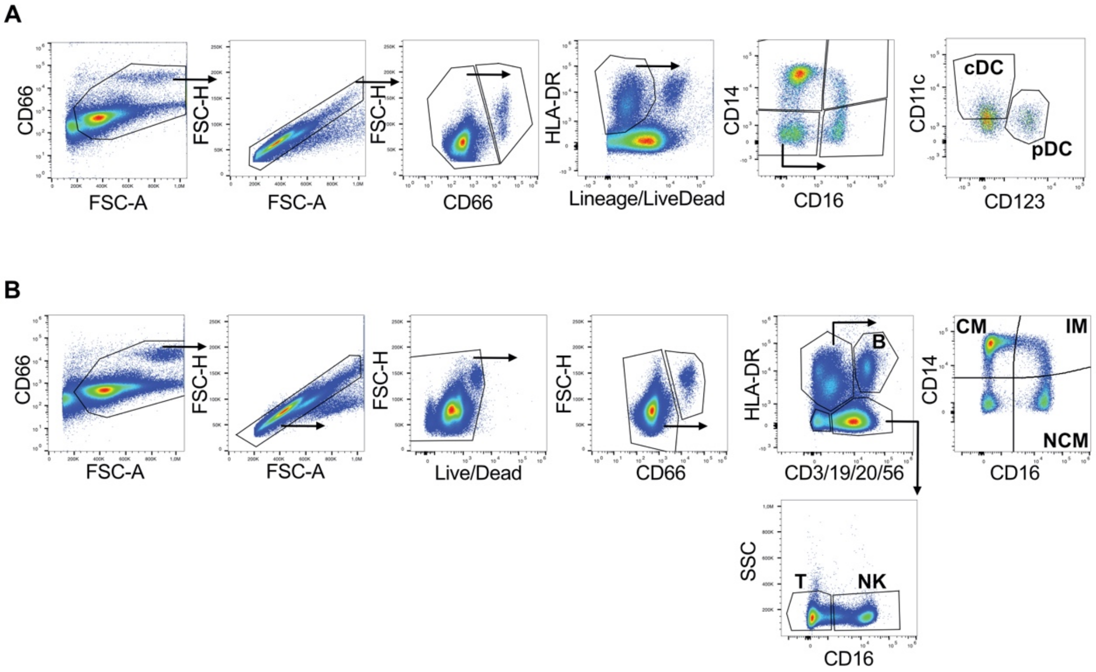

Figure S13

Figure S13: Gating strategies for innate immune cell populations. Representative gating strategies used to identify dendritic cell populations **(A),** and monocyte and lymphocyte subpopulations **(B).** cDC: Conventional dendritic cells. pDC: Plasmacytoid dendritic cells. B= B cells. T = T cells. NK = Natural Killer cells. CM = Classical monocytes. IM = Intermediate monocytes. NCM = Non-classical monocytes. Time gates were applied where required for technical reasons.

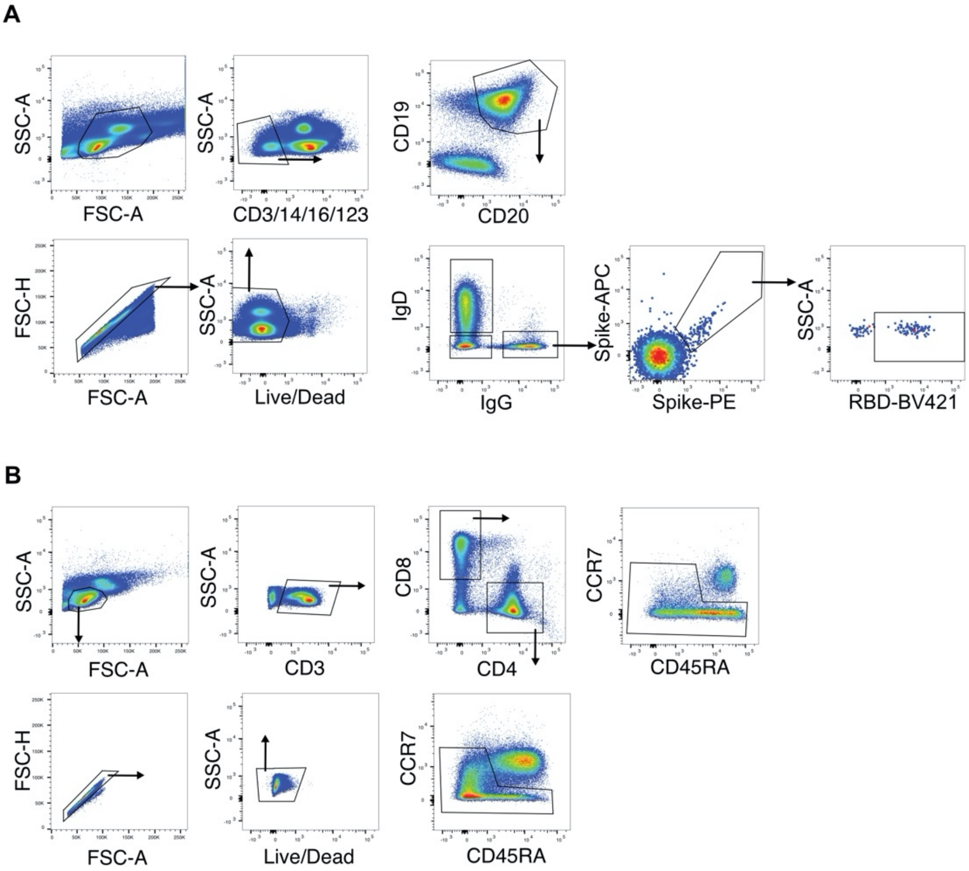

Figure S14

Figure S14: Gating strategies for adaptive cellular responses. A: Representative gating strategy used to identify SARS-CoV-2 Spike and RBD-specific IgG memory B cells using fluorescent probe staining. B: Representative gating strategy used to identify CD4 and CD8 memory T cells. Example gating for cytokine-producing CD69+. A small subset of samples were stained using LiveDead Aqua rather than Live/Dead Blue for technical reasons. Time gates were applied where required for technical reasons.
